# Supplementary material for: Heavy khat (Catha edulis) chewing and dyslipidemia as modifiable hypertensive risk factors among patients in Southwest, Ethiopia: Unmatched case-control study
Source: PLoS One. 2021 Oct 26;16(10):e0259078. doi: 10.1371/journal.pone.0259078 (PMC8547649; doi:10.1371/journal.pone.0259078)
Supplement: S1 Questionnaire — (DOCX) [file pone.0259078.s001.docx]

**Appendix I: - English Questionnaires**

Hello! My name is ________and I am collecting data for a study on the *heavy khat (Catha edulis) chewing and dyslipidemia as modifiable hypertensive risk factors among patients in Southwest, Ethiopia.* I would like to ask you some questions about the risk factors some risk factors of hypertension and if you are willing I will also take some physical measurements and blood sample. The interview would take about 25 minutes. By going through the study you will know your lipid profile, blood pressure and other anthropometric parameters of yourself. Your participation in the study is very critical for developing an input for assessing risk factors of hypertension for hospital community as well as for Jimma Zone, too. However, your participation is entirely based on your willingness and your refusal doesn’t affect the service you get from us in any way. The data you provided will be kept in highly confidential manner and none of your personal identifiers will be on the questionnaire. If you want to know your results you can check it yourself using your secret Identification number that we shall provide you. All people analyzing the laboratory results and the data itself do not have access to your personal identifiers.

Verbal consent obtained Yes
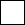
 No
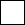


Signature

Date of data collection _____ / _____ / ______

(Ethiopian calendar: Day Month Year)

**PART I: Questions on Socio-economic and Socio- demographic characteristics of the respondent**

| **No.** | **Questions** | **Response categories** | **Remarks** |
| --- | --- | --- | --- |
| 101 | Identification Number | ID NO ----------------------- |  |
| 102 | Sex | 1. Male 2. Female |  |
| 103 | Age of respondent | In competed years ------------ |  |
| 104 | Where is your residence? | 1. Urban ------------------ 2. Rural ------------------- |  |
| 105 | What is your ethnicity? | 1. Oromo 2. Amhara 3. Dawuro 4. Kafa 5. Yem 6. Other(specify) |  |
| 106 | What is your marital Status? | 1. Married 2. Single 3. Divorced 4. Widowed 5. Separated |  |
| 107 | What is your religion? | 1. Orthodox 2. Muslim 3. Protestant 4. Catholic 5. Other (specify) |  |
| 108 | Educational status of the respondent | 1. No formal education 2. Primary educational level (1-8) 3. Secondary and above (above grade 9) |  |
| 109 | What is your occupation? | 1. Farmer 2. Government Employee 3. Student 4. House wife 5. Jobless(dependent) 6. Self-Employ 7. Other (specify) -------- |  |
| 110 | If ye for 102, do you have history of Oral contraceptive use? | 1. Yes 2. No |  |
| 111 | Do you have previous history of hypertension? | 1. Yes 2. No |  |
| 112 | Do you have family history of hypertension? | 1. Yes 2. No |  |
| 113 | How much money do you earn on monthly basis | -------------------------- |  |
| 114 | What is the monthly income of your house hold (total income)? | _____________Ethiopian birr |  |

**PART II: BEHAVIOURAL MEASUREMENTS**

| **Tobacco use practice** | | | | | | |
| --- | --- | --- | --- | --- | --- | --- |
| **No.** | | **Questions** | | **Response categories** | | **Remarks** |
| 201 | | Do you have history of cigarettes smoking? | | 1. Yes 2. No | |  |
| 202 | | If yes to 201, how often were you smoking? | | 1. Daily 2. 3 times per week 3. Once a week 4. Once a month | |  |
| 203 | | If Yes to 201, how long ago did you start smoking? | |  | |  |
| 204 | | On average, how many of the cigarettes do you smoke each day/week? | |  | |  |
| 205 | | Do you currently smoke tobacco products **daily**? | | 1. Still smoking 2. Reduced 3. Ceased | |  |
| 206 | | Is there family member who smoke cigarette? | | 1. Yes 2. No | |  |
| 207 | | How often was He/She smoking? | | 1. Daily 2. 3 times per week 3. Once a week 4. Once a month | |  |
| **Alcohol use practice** | | | | | | |
| 208 | Do you have history of alcohol drinking? | | 1. Yes 2. No | |  | |
| 209 | If yes to 305, which one? | | 1. Beer/Draft 2. Wine 3. Tejj 4. Local areke 5. Others (specify) | |  | |
| 210 | **How frequently** have you had at least one standard alcoholic drink? | | 1. Daily 2. 5-6 days per week 3. 3-4 days per week 4. 1-2 days per week 5. 1-3 days per month 6. Less than once a month | |  | |
| 211 | When you drank alcohol, how many standard **drinks on average** did you have during one drinking occasion? | | **___________________Drinks** | |  | |
| 212 | Are you currently drinking alcohol daily? | | 1. Still drinking 2. Reduced 3. Ceased | |  | |
| **KHAT Chewing** | | | | | | |
| 213 | Do you chew Khat? | | 1. Yes 2.No | |  | |
|  | Frequency of chewing | | 1. Daily 2. 3 times per week 3. Once a week 4. Once a month | |  | |
| 214 | If Yes, how often do you chew Khat? | | 1. Daily 2. 3 times per week 3. Once a week 4. Once a month | |  | |
| 215 | How many **zurbas** do you chew on **one** of those days? | | ----------------zurbas | |  | |
| 216 | Reason of chewing khat? | |  | |  | |
| **Physical Inactivity** | | | | | | |
| 217 | Do you do any vigorous-intensity sports, fitness or recreational (leisure) activities that cause large increases in breathing or heart rate like [running or football] for at least 75–150 minutes per week continuously? | | 1. Yes 2. No | |  | |
| 218 | Do you do any moderate-intensity sports, fitness or recreational (leisure) activities that cause large increases in breathing or heart rate like [running or football] for at least 150-300 minutes per week continuously? | | 1. Yes 2. No | |  | |

**Part III: Dietary practice**

| **No.** | **Questions** | **Response categories** | **Remarks** |
| --- | --- | --- | --- |
| 301 | In a typical week, on how many days do you **eat fruit**? | __________________**Days** |  |
| 302 | How many **servings** of fruit do you eat on **one** of those days? | ______________**Servings**(a serving in this case refers to one fruit eg.orange, banana, mango...) |  |
| 303 | In a typical week, on how many days do you **eat vegetables**? | **_________________Days** |  |
| 304 | How many **servings** of vegetables do you eat on one of those days? | _______________**Servings**(a serving in this case refers cups of vegetable stews...) |  |
| 305 | What type of **oil or fat is most often** used for meal preparation in your household?  **(*Circle ONLY ONE answer)*** | 1. Liquid Vegetable oil 2. Solidified (palm) oil 3. Butter 4. Margarine / peanut butter 5. Sheno lega 6. Other |  |
| 306 | Amount of salt you consume | 1. High 2. Optimal |  |

**Part IV: Physical measurements**

| **Blood Pressure(BP)(mmHg)** | | | | |
| --- | --- | --- | --- | --- |
| 401 | Cuff Size used | **1.Small**  **2.Medium**  **3.Large** | |  |
| 402 | Reading 1 | **Systolic(mmHg)_____________**  **Diastolic(mmHg)___________** | |  |
|  | Reading 2 | **Systolic(mmHg)_____________**  **Diastolic(mmHg) ___________** | |  |
|  | Reading 3 | **Systolic(mmHg)_____________**  **Diastolic(mmHg) ___________** | |  |
| **Anthropometry Measurements** | | | | |
| 403 | Height | Height in(m)______________ |  | |
| 404 | Weight | Weight (kg)__________ |  | |
| 405 | BMI | __________ |  | |
| **Biochemical measurements** | | | | |
| 406 | Total Cholesterol | Chol.(mg/dl)__________ |  | |
| 407 | High density Lipoprotein(HDL)(mg/dl) | HDL(mg/dl)_____________ |  | |
| 408 | Low density Lipoprotein (LDL ) | LDL(mg/dl)__________ |  | |
| 409 | Triglycerides (TG) | TG(mg/dl) __________ |  | |
