# Supplementary material for: Heavy khat (Catha edulis) chewing and dyslipidemia as modifiable hypertensive risk factors among patients in Southwest, Ethiopia: Unmatched case-control study
Source: PLoS One. 2021 Oct 26;16(10):e0259078. doi: 10.1371/journal.pone.0259078 (PMC8547649; doi:10.1371/journal.pone.0259078)
Supplement: S2 Questionnaire — (DOCX) [file pone.0259078.s002.docx]

**Appendix II: - Amharic Questionnaires**

**አባሪ: - መጠይቆች**

ሰላም ነው! እኔ ________ እባላለው፡፡ በከባድ ጫት መቃም እና ያላግባብ በሰውነት ውስጥ የተጠራቀመ ስብ ምክንያት እና ሌሎች አጋላጭ የደም ግፊት ሊያመጡ የሚችሉ ምክንያቶች ላይ ጥናት ለማድረግ መረጃዎችን እየሰበሰብኩ እገኛለው ስለሆነም እርሶ ፍቃደኛ ከሆኑ እኔ አንዳድ ጥያቄዎችን አቀረባለው፡፡

ስለ አንዳንድ የደም ግፊት አደጋ ተጋላጭነት ምክንያቶች አንዳንድ ጥያቄዎችን ልጠይቃችሁ እወዳለሁ እናም ፈቃደኛ ከሆኑ የተወሰኑ የሰውነት መለኪያዎች እና የደም ናሙናም እወስዳለሁ ፡፡ ቃለመጠይቁ 25 ደቂቃ ያህል ይወስዳል ፡፡ በጥናቱ ውስጥ በማለፍ የሊፕቲድ ፕሮፋይልዎን (የደም ውስጥ የስብ መጠኖን)፣ የደም ግፊትዎን እና ሌሎች የሰውነት ልኬቶች ያውቃሉ ፡፡ ለሆስፒታሎች ማህበረሰብ እንዲሁም ለጅማ ዞን የደም ግፊት ተጋላጭነት ሁኔታዎችን ለመገምገም ግብዓት ለማዘጋጀት ጥናቱ ውስጥ የእርስዎ ተሳትፎ በጣም ወሳኝ ነው ፡፡ ሆኖም የእርስዎ ተሳትፎ ሙሉ በሙሉ በፈቃደኝነትዎ ላይ የተመሠረተ ነው እናም እምቢታዎ በምንም መንገድ ከእኛ በሚያገኙት አገልግሎት ላይ ተጽዕኖ የለውም ፡፡ ያቀረቡት መረጃ በከፍተኛ በሚስጥራዊ ሁኔታ ይቀመጣል ፣ እና የግል መለያዎቻችሁም በመጠይቁ ላይ አይሆኑም። ውጤቶችዎን ማወቅ ከፈለጉ እኛ ለእርስዎ የምናቀርበውን የምስጢር መለያ ቁጥርዎን በመጠቀም እራስዎ ማረጋገጥ ይችላሉ ፡፡ የላብራቶሪ ውጤቶችን እና መረጃውን የሚተነትኑ ሰዎች ሁሉ የግል መለያዎችዎ መዳረሻ የላቸውም።

የቃል ስምምነት ተገኝቷል አዎ የለም

ፊርማ

የመረጃ መሰብሰቢያ ቀን _____ / _____ / ______

(የኢትዮጵያ አቆጣጠር የቀን ወር ዓመት)

ክፍል I: በተጠየያሪ ማህበራዊ-ኢኮኖሚያዊ እና ማህበራዊ-ስነ-ህዝብ ባህሪዎች ላይ ጥያቄዎች

| ተራ ቁ | ጥያቄዎች | የምላሽ ምድቦች | አስተያየቶች |
| --- | --- | --- | --- |
| 101 | መታወቂያ ቁጥር |  |  |
| 102 | ፆታ | 1. ወንድ 2. ሴት |  |
| 103 | ዕድሜ | ሙሉ ቁጥር |  |
| 104 | መኖሪያዎ የት ነው? | ከተማ ------------------  2. ገጠር -------------- |  |
| 105 | ጎሳህ ምንድነው? | 1. ኦሮሞ  2. አማራ  3. ዳውሮ  4. ካፋ  5. ኢም  6. ሌላ (ይግለጹ) |  |
| 106 | የጋብቻ ሁኔታዎ | 1. ያገባ  2. ያላገባ  3. የተፋታ  4. መበለት  5. ተለያይቷል |  |
| 107 | ሃይማኖትህ ምንድነው? | 1. ኦርቶዶክስ  2. ሙስሊም  3. ፕሮቴስታንት  4. ካቶሊክ  5. ሌላ (ይግለጹ) |  |
| 108 | የትምህርት ደረጃ | 1. መደበኛ ትምህርት የለም  2. የመጀመሪያ ደረጃ ትምህርት ደረጃ (1-8)  3. ሁለተኛ እና ከዚያ በላይ (ከ 9 ኛ ክፍል በላይ) |  |
| 109 | ሥራዎ ምንድነው? | 1. ገበሬ  2. የመንግስት ሰራተኛ  3. ተማሪ  4. የቤት ሚስት  5. ሥራ አጥ (ጥገኛ)  6. በራስ ሥራ መሥራት  7. ሌላ (ይግለጹ) ------- |  |
| 110 | ለጥያቄ ቁጥር 102 አዎ ከሆነ በአፍ የሚወሰድ እንክብል የወሊድ መከላከያ ተጠቅሞ የውቃሉ | 1. አዎ  2. የለም |  |
| 111 | ከዚህ በፊት የደም ግፊት ታሪክ አለዎት? | 1. አዎ  2. የለም |  |
| 112 | የደም ግፊት በቤተሰቦ አለዎት? | 1. አዎ 2. አይ |  |
| 113 | በየወሩ ምን ያህል ገንዘብ ያገኛሉ | _____________ የኢትዮጵያ ብር |  |
| 114 | የቤትዎ ወርሃዊ ገቢ (ጠቅላላ ገቢ) ስንት ነው? | _____________ የኢትዮጵያ ብር |  |

ክፍል II-የባህርይ መለኪያዎች

| የትምባሆ አጠቃቀም ልምድ | | | |
| --- | --- | --- | --- |
|  | ጥያቄዎች | የምላሽ ምድቦች | አስተያየቶች |
| 201 | ሲጋራ የማጨስ ታሪክ አለዎት? | 1. አዎ 2. አይ |  |
| 202 | ለ 201 አዎ ከሆነ ስንት ጊዜ ያጨሱ ነበር? | 1. በየቀኑ  2. በሳምንት 3 ጊዜ  3. በሳምንት አንድ ጊዜ  4. በወር አንድ ጊዜ |  |
| 203 | ለ 201 አዎ ከሆነ ስንት ጊዜ ሆነው ማጨስ ከጀመሩ? |  |  |
| 204 | በአማካይ በየቀኑ / በሳምንት ስንት ሲጋራ ታጨሳለህ? |  |  |
| 205 | በአሁኑ ጊዜ የትምባሆ ምርቶችን በየቀኑ ያጨሳሉ? | 1. አሁንም ማጨስ  2. ቀንሷል  3. ተቋርጧል |  |
| 206 | ሲጋራ የሚያጨስ አንድ የቤተሰብ አባል አለ? | 1. አዎ 2. አይደለም |  |
| 207 | እሱ / እሷ ስንት ጊዜ ያጨስ ነበር? | 1. በየቀኑ  2. በሳምንት 3 ጊዜ  3. በሳምንት አንድ ጊዜ  4. በወር አንድ ጊዜ |  |
| የአልኮሆል አጠቃቀም ልምምድ | | | |
| 208 | የመጠጥ ታሪክ አለዎት? | 1. አዎ 2. አይደለም |  |
| 209 | አዎ ከሆነ 305 ከሆነ የትኛው ነው? | 1. ቢራ 2. ሀረቄ   2. ወይን  3. ተጃ  4. አካባቢያዊ አረክ  5. ሌሎች (ይግለጹ) |  |
| 210 | ቢያንስ አንድ መደበኛ የአልኮሆል መጠጥ ምን ያህል ጊዜ ጠጥተዋል? | 1. በየቀኑ  2. በሳምንት 5-6 ቀናት  3. በሳምንት 3-4 ቀናት  4. በሳምንት 1-2 ቀናት  5. በወር ከ1-3 ቀናት  6. በወር ከአንድ ጊዜ በታች |  |
| 211 | አልኮል ሲጠጡ በአንድ የመጠጥ ወቅት በአማካይ ምን ያህል መደበኛ መጠጦች ነዎት? |  |  |
| 212 | በአሁኑ ጊዜ በየቀኑ አልኮል እየጠጡ ነው? | 1. አሁንም መጠጣት  2. ቀንሷል  3. ተቋርጧል |  |
| ጫት መቃም | | | |
| 213 | ጫት ይቅማሉ | 1. አዎ 2. አይደለም |  |
| 214 | አዎ ከሆነ ስንት ጊዜ ጫት ይቅማሉ? | 1. በየቀኑ  2. በሳምንት 3 ጊዜ  3. በሳምንት አንድ ጊዜ  4. በወር አንድ ጊዜ |  |
| 215 | ከነዚህ ቀናት በአንድ ጊዜ ስንት ዙርባዎችን ይቅማሉ? | 1. ከአንድ በታች 2. አንድ 3. ከአንድ በላይ |  |
| 216 | ጫት የመቃም ምክንያት? |  |  |
| አካላዊ እንቅስቃሴ | | | |
| 217 | ያለማቋረጥ በሳምንት ቢያንስ ለ 75-150 ደቂቃዎች እንደ [ሩጫ ወይም እግር ኳስ] እንደ መተንፈስ ወይም የልብ ምት ከፍተኛ ጭማሪን የሚያመጡ ኃይለኛ ስፖርቶችን ፣ የአካል ብቃት እንቅስቃሴዎችን ወይም መዝናኛ (መዝናኛ) እንቅስቃሴዎችን ያደርጋሉ? | 1. አዎ 2. አይደለም |  |
| 218 | ያለማቋረጥ በሳምንት ቢያንስ ከ150-300 ደቂቃዎች ያህል [እንደ ሩጫ ወይም እግር ኳስ] እንደ መተንፈስ ወይም የልብ ምት ከፍተኛ ጭማሪ የሚያስከትሉ መጠነኛ ኃይለኛ ስፖርቶችን ፣ የአካል ብቃት እንቅስቃሴዎችን ወይም መዝናኛ (መዝናኛ) እንቅስቃሴዎችን ያደርጋሉ? | 1. አዎ 2. አይደለም |  |
| ክፍል III: - የአመጋገብ ልምምድ | | | |
| 301 | በተለመደው ሳምንት ውስጥ ስንት ቀናት ፍሬ ይበላሉ? | __________________ ቀናት |  |
| 302 | በእነዚያ ቀናት በአንዱ ስንት ፍሬዎችን ይመገባሉ? | ______________ አገልግሎት (በዚህ ጉዳይ ላይ አገልግሎት መስጠት አንድ ፍሬ ለምሳሌ ብርቱካናማ ፣ ሙዝ ፣ ማንጎ ...) |  |
| 303 | በተለመደው ሳምንት ውስጥ ስንት ቀናት አትክልቶችን ይመገባሉ? | _________________ ቀናት |  |
| 304 | በእነዚያ ቀናት በአንዱ ስንት አትክልቶችን ይመገባሉ? _______________ አገልግሎቶች (በዚህ ጉዳይ ላይ አንድ አገልግሎት የአትክልት ስኒዎችን ኩባያ ያመለክታል ...) |  |  |
| 305 | አብዛኛውን ጊዜ በቤተሰብዎ ውስጥ ለምግብ ዝግጅት የሚውለው ምን ዓይነት ዘይት ወይም ስብ ነው? | 1. ፈሳሽ የአትክልት ዘይት  2. የተጠናከረ (የዘንባባ) ዘይት  3. ቅቤ  4. ማርጋሪን / የኦቾሎኒ ቅቤ  5. ሸኖ ለጋ  6. ሌላ |  |
| 306 | የሚወስዱት የጨው መጠን | 1. ከፍተኛ  2. ምርጥ |  |
| ክፍል IV: አካላዊ መለኪያዎች | | | |
| 401 | የደም ግፊት (ቢፒ) (mmHg) | 1. አነስተኛ  2. መካከለኛ  3. ትልቅ |  |
| 402 | ሲስቶሊክ (mmHg) _____________ | ንባብ1  ንባብ1  ንባብ1 |  |
| 403 | ዲያስቶሊክ (mmHg) ___________ |  |  |
| 404 | ቁመት ቁመት በ (m) ______________ |  |  |
| 405 | ክብደት ክብደት (ኪግ) __________ |  |  |
| 406 | ቁመት |  |  |
|  | BMI |  |  |
| ክፍል IV: ባዮኬሚካላዊ መለኪያዎች | | | |
| 312 | Total Cholesterol | Chol.(mg/dl)__________ |  |
| 313 | High density Lipoprotein(HDL)(mg/dl) | HDL(mg/dl)_____________ |  |
| 314 | Low density Lipoprotein (LDL ) | LDL(mg/dl)__________ |  |
| 315 | Triglycerides (TG) | TG(mg/dl) __________ |  |
