# Supplementary material for: Heavy khat (Catha edulis) chewing and dyslipidemia as modifiable hypertensive risk factors among patients in Southwest, Ethiopia: Unmatched case-control study
Source: PLoS One. 2021 Oct 26;16(10):e0259078. doi: 10.1371/journal.pone.0259078 (PMC8547649; doi:10.1371/journal.pone.0259078)
Supplement: S3 Questionnaire — (DOCX) [file pone.0259078.s003.docx]

**Appendix III: - Oromiffa Questionnaires**

**Kutaa I: Gaaffilee haala jiruuf jireenyaa,hawaasummaa fi qabeenyaa Gaafatamaa**

| **Lakk** | **Gaaffilee** | **Gaaaffilee** | **Yaadachiisa** |
| --- | --- | --- | --- |
| 101 | Lakkoofsa addaa | Lakk ----------------------- |  |
| 102 | Saala | 1. Dhiira 2.Dhalaa |  |
| 103 | Umurii | Waggaadhaan------------ |  |
| 104 | Bakki jireenya kee essa? | 1. Magaalaa----------------- 2. Baadiyaa------------------- |  |
| 105 | Sabni kee maalii? | 1. Oromoo 2. Amaara 3. Daawuroo 4. Kafaa 5. Yeem 6. Kan biraa(ibsii) |  |
| 106 | Haalaa gaa’elaa? | 1. Fuudheera 2. Kan hin fuune 3. Kan hike/te(karaa seeraa) 4. Kan irraa du’e 5. Kan hike/te(seeraa ala) |  |
| 107 | Amantiin kee malii? | 1. Ortoodoksii 2. Musliima 3. Pirootestaantii 4. Kaatolikii 5. Kan biraa (Ibsi) |  |
| 108 | Sadarkaan barnoota kee hagami? | 1. Barnoota hin baranne 2. Barnoota sadarkaa tokkoffaa(1-8) 3. Barnoota sadarkaa lammaffaa fi isaa ol |  |
| 109 | Hojiin kee maali? | 1. Qotee bulaa 2. Hojjetaa mootummaa 3. Barataa 4. Haadha warraa 5. Hojii dhabaa 6. Hojii dhuunfaa 7. Hojii kan biraa(ibsi) |  |
| 110 | Kininii ulfaa ittisu kan liqimsaa fudhattee beektaa? | 1. Eeyyee 2. Lakkii |  |
| 111 | Dhukkuba dhiibbaa dhiigaa kanaan dura qabdaa? | 1. Eyyee 2. Lakkii |  |
| 112 | Maatiikee keesaa namni dhiibbaa dhiigaa qabu ni jiraa? | 1. Eeyyee 2. Lakkii |  |
| 113 | Ji’aan galii qarshii hagam argatta? | -------------------------- |  |
| 114 | Maatiin kee walumaa galatti Ji’aan galii hangam argatu (Galii walii galaa)? | _____________qarshii itoophiyaa. |  |

**Kutaa II: Safartoolee amalootaa**

| **Tamboo xuuxuu** | | | | | | |
| --- | --- | --- | --- | --- | --- | --- |
| **Lakk.** | | **Gaaffilee** | | **Deebii** | | **Yaadachiisa** |
| 201 | | Kanaan dura tamboo xuuxxee ni beektaa? | | 1. Eeyee 2. lakkii | |  |
| 202 | | Yoo deebiin kee gaaffii 201f eeyyee tahe, hammam hammamiin xuuxxaa? | | 1. Guyyaan 2. Toorbanitti guyyaa sadii 3. Toorbaniti guyyaa tokko 4. Ji’atti guyyaa tokko | |  |
| 203 | | Yoo deebiin kee gaaffii 201f eeyyee tahe, yoom tamboo xuuxuu eegalte? | |  | |  |
| 204 | | Tilmaamaan guyyaa guyyaan timboo meeqa xuuxxa toorbanitti | |  | |  |
| 205 | | Amma guyyaa guyyaadhaan tamboo xuuxaa jirtaa?? | | 1. Ammayyu xuuxaan n jira 2. Hir’iseera 3. dhiiseera | |  |
| 206 | | Maatii kee keessaa namni biraa tamboo xuuxu ni jiraa?? | | 1. Eeyee 2. lakkii | |  |
| 207 | | Hagam hagamiin xuuxaa/ti? | | 1. Guyyaan 2. Toorban keessatti guyaa sadii 3. Toorbanitti guyyaa tokko 4. Ji’atti guyyaa tokko | |  |
| **Dhugaatii alkoolii** | | | | | | |
| 208 | Kanaan dura alkoolii dhugdee beektaa? | | 1. Eeyyee 2. Lakkii | |  | |
| 209 | Yoo deebiin kee gaaffii 208f eeyyee tahe, alkoolii kamii? | | 1. Biiraa 2. Wayinii 3. daadhii 4. Araqee 5. Kan biraa(ibsi) | |  | |
| 210 | **Yeroo hangam hangamitti alkoolii dhugda(tokkos tahu**? | | 1. Guyyaa Guyyaan 2. Guyyaa 5-6 toorban keessatti 3. Guyyaa 3-4 toorbanitti 4. Guyyaa 1-2 toorbanitti 5. Guyyaa 1-2 ji’a keesattti 6. Guyaa 1 gadi ji’a keessatti | |  | |
| 211 | Yeroo dhugaatii alkoolii dhugduu al takkaan hangam dhugdaa?? | | **___________________Dhugaatii** | |  | |
| 212 | Ammayyu alkoolii guyyaa guyyaan dhugaa jirtaa? | | 1. Ammayyu dhugaan jira 2. Hir’iseera 3. dhaaabeera | |  | |
| **CAATII QAMA’UU** | | | | | | |
| 213 | Caatii ni qamaataa? | | 1. Eeyyee 2.Lakkii | |  | |
| 214 | Yoo deebiin kee gaaffii 213f eeyyee tahe, Yoom yoom qamaata? | | 1. Guyyaan 2. Toorbanitti guyyaa sadii 3. Toorbaniti guyyaa tokko 4. Ji’atti guyyaa tokko | |  | |
| 215 | Yeroo tokkoon qabaa meeqa qamaata? | | ----------------/qaba | |  | |
| 216 | Sababa maaliif qamaata? | |  | |  | |
| **Sosochii qaamaa gochuu dhiisuu** | | | | | | |
| 217 | Sosochii qaamaa fi jajjabina qaamaa kanneen akka ispoortii,jajjabina qaamaa fi bashannanaa kanneen hargansuu fi dha’annaa onnee dabalan(fiiguu fi kubbaa miilaa) yoo xiqqaatee toorbanitti daqiiqaa 75-150f walitti fufinsaan ni gootaa? | | 1. Eeyyee 2. Lakkii | |  | |
| 218 | Sosochii qaamaa fi jajjabina qaamaa giddu galeessaa fi ulfaatookanneen akka ispoortii,,jajjabina qaamaa fi bashannanaa kanneen hargansuu fi dha’annaa onnee hedduumminaan dabalan(fiiguu fi kubbaa miilaa) yoo xiqqaatee toorbanitti daqiiqaa 150-300f walitti fufinsaan ni gootaa? | | 1. Eeyyee 2. **Lakkii** | |  | |

**Kutaa III: Amaloota soorataa**

| **Lakk** | **Gaaffii** | **Deebii gaaffilee** | **Yaadachiisa** |
| --- | --- | --- | --- |
| 301 | Toorban keesastti guyyaa meeqa fuduraa soorattaa? | Guyyoota__________________ |  |
| 302 | Fuduraalee nyaattu kaneen guyaatti ija meeqa sooratta? | Ija_____________**(**Burtukaanii,muuzii, maangoo) |  |
| 303 | Toorban keessatti guyyaa meeqa muduraalee sooratta? | **Guyyaa _________________** |  |
| 304 | Muduraalee soorattu kanneen guyyaatti ija meeqa sooratta? | _______________**guyyoota fayyadamtu kanneeni keessaa guyyaa tokkotti hanga fayyadamtu safaraan ibsi**.) |  |
| 305 | Mana keessan keessati soorata bilcheessuuf coomaa fi zayitii gosa kam fayyadamtan?  **Deebii tokko qofaatti mari*)*** | 1. Zayitii dhangala’aa 2. Zayittii jajjaboo(palm oil) 3. Dhadhaa 4. Dhadhaa margaarinii( peanut butter) 5. Dhadhaa shanoo 6. Kannen biroo |  |
| 306 | Soogidda hangam soorattan? | 1. Baayyee 2. Giddu galeessa |  |

**Kutaa IV: Safartoota fiizikaalaa**

| **Dhiibaa dhiigaa (BP in mmHg)** | | | | |
| --- | --- | --- | --- | --- |
| 401 | Hamma cuff fayyadamtee | **1.Xiqqoo**  **2.Giddu galeessa**  **3.Gudda** | |  |
| 402 | Safara 1ffaa | **Siistoolikii (mmHg)_____________**  **Diyaastoolikii(mmHg)___________** | |  |
|  | Safara 2ffaa | **Siistoolikii(mmHg)_____________**  **Diyaastoolikii(mmHg) ___________** | |  |
|  | Safara 3ffaa | **Siistoolikii(mmHg)_____________**  **Diyaastoolikii(mmHg) ___________** | |  |
| **Safartoota Antirooppoomeetirii** | | | | |
| 403 | Dheerina | Dheerina (cm)______________ |  | |
| 404 | Ulfaatina | Ulfaatina(kg)__________ |  | |
| 405 | BMI | __________kg/m2 |  | |
| **Safartoota baayoomeedikaalii** | | | | |
| 406 | Total cholesterol | Chol.(mg/dl)__________ |  | |
| 407 | High density Lipoprotein(HDL)(mg/dl) | HDL(mg/dl)_____________ |  | |
| 408 | Low density Lipoprotein (LDL ) | LDL(mg/dl)__________ |  | |
| 409 | Triglycerides (TG) | TG(mg/dl) __________ |  | |
