# Supplementary material for: Heavy khat (Catha edulis) chewing and dyslipidemia as modifiable hypertensive risk factors among patients in Southwest, Ethiopia: Unmatched case-control study
Source: PLoS One. 2021 Oct 26;16(10):e0259078. doi: 10.1371/journal.pone.0259078 (PMC8547649; doi:10.1371/journal.pone.0259078)
Supplement: Attachment — Submitted filename: PONE-D-20-40692_reviewer [Khat_&_Hypertension].pdf [file pone.0259078.s005.pdf]

PLOS ONE

Heavy khat (Catha edulis) chewing and dyslipidemia as a cardiovascular risk factor among hypertensive patients in Jimma, Ethiopia: Unmatched case control study

--Manuscript Draft--

**Manuscript Number:**	PONE-D-20-40692
**Article Type:**	Research Article
**Full Title:**	Heavy khat (Catha edulis) chewing and dyslipidemia as a cardiovascular risk factor among hypertensive patients in Jimma, Ethiopia: Unmatched case control study
**Short Title:**	Heavy khat (Catha edulis) chewing and dyslipidemia as a cardiovascular risk factor
**Corresponding Author:**	Meron Hadis Gebremedhin, MSc<br>Wolaita Sodo University<br>Wolaita Sodo, ETHIOPIA
**Keywords:**	Keywords: Dyslipidemia; khat chewing; Hypertension; Case-control
**Abstract:**	Abstract<br>Background: Hypertension is a state of high blood pressure; among the cardiovascular disease risk factors dyslipidemia and khat chewing were increasing at an alarming rate in Ethiopia but still neglected. This study aimed to assess heavy khat (Catha edulis) chewing and dyslipidemia as a cardiovascular risk factor among hypertensive patients in Southwest Ethiopia.<br>Methods: A facility-based case-control study was conducted on hypertensive and non-hypertensive individuals recruited from the chronic illness clinic of Jimma University medical center. The consecutive sampling technique was employed, and data were collected from May 15 to July 30, 2017. The data were entered using Epi data version 3.1 and exported to SPSS Version 21 for analysis. Logistic regression was performed, and statically significant were considered a cardiovascular risk factor among hypertensive patients.<br>Results: Of the 406 participants, 136 hypertensive cases and 270 non-hypertensive controls were involved. Most were TC/HDL ratio of >5 (72.1%) and (23.7%) of controls had dyslipidemia. Among Khat chewers, 54.67% had dyslipidemia; 72.9% use packed oils for food preparation of which 72.5% had dyslipidemia. Educational status (7.33; 2.66-20.19), excess alcohol consumption (3.33; 1.69-6.57), moderate khat chewers (3.54; 1.83-6.84), heavy khat chewers (19.12; 4.62-79.06), and vigorous work-related activity (3.84; 1.95-7.53), low HDL level (0.56; 0.32-0.97), dyslipidemia (11.83; 6.71-20.87) increases the odds of hypertension.<br>Conclusion: In this study, dyslipidemia and heavy khat chewing were statistically associated. And both are the major risk factors. Therefore, due consideration should be given to prevent and control risk factors by designing health information provision systems; promoting acceptable health practices. It should give particular attention to those growing farmers to minimize khat cultivation and awareness creation to the use of khat and increase educational attainment, change related behavioral factors, decrease work-related stress and focus on the type of oil.<br>Keywords: Dyslipidemia, Khat chewing, Hypertension, Case-control
**Order of Authors:**	Meron Hadis Gebremedhin, MSc
**Additional Information:**	
**Question**	**Response**
**Financial Disclosure**<br><br>Enter a financial disclosure statement that describes the sources of funding for the work included in this submission. Review the submission guidelines for detailed requirements. View published research articles from *PLOS ONE* for specific	No funding received but Jimma University paid per diam for data collectors

Powered by Editorial Manager® and ProduXion Manager® from Aries Systems Corporation

examples.<br><br>This statement is required for submission and **will appear in the published article** if the submission is accepted. Please make sure it is accurate.<br><br>**Unfunded studies**<br>Enter: *The author(s) received no specific funding for this work.*<br><br>**Funded studies**<br>Enter a statement with the following details:<br>• Initials of the authors who received each award<br>• Grant numbers awarded to each author<br>• The full name of each funder<br>• URL of each funder website<br>• Did the sponsors or funders play any role in the study design, data collection and analysis, decision to publish, or preparation of the manuscript?<br>• **NO** - Include this sentence at the end of your statement: *The funders had no role in study design, data collection and analysis, decision to publish, or preparation of the manuscript.*<br>• **YES** - Specify the role(s) played.<br><br>* typeset	
**Competing Interests**<br><br>Use the instructions below to enter a competing interest statement for this submission. On behalf of all authors, disclose any competing interests that could be perceived to bias this work—acknowledging all financial support and any other relevant financial or non-financial competing interests.<br><br>This statement **will appear in the published article** if the submission is accepted. Please make sure it is accurate. View published research articles from *PLOS ONE* for specific examples.	No competing interests.

Powered by Editorial Manager® and ProduXion Manager® from Aries Systems Corporation

**NO authors have competing interests**<br><br>Enter: *The authors have declared that no competing interests exist.*<br><br>**Authors with competing interests**<br><br>Enter competing interest details beginning with this statement:<br><br>*I have read the journal's policy and the authors of this manuscript have the following competing interests: [insert competing interests here]*<br><br>* typeset	
**Ethics Statement**<br><br>Enter an ethics statement for this submission. This statement is required if the study involved:<br><br>• Human participants<br>• Human specimens or tissue<br>• Vertebrate animals or cephalopods<br>• Vertebrate embryos or tissues<br>• Field research<br><br>Write "N/A" if the submission does not require an ethics statement.<br><br>General guidance is provided below. Consult the submission guidelines for detailed instructions. **Make sure that all information entered here is included in the Methods section of the manuscript.**	The study was approved by Jimma University institute of health science and a support letter was obtained from the department of biomedical science and Internal medicine ethical review committee. All the study participants were informed about the purpose of the study, their right to refuse and assured confidentiality and informed verbal and written consent was obtained prior to the interview. At the end of each interview and measurement procedures, awareness creation was made to study participants in control groups about the risk factors of cardiovascular disease and hypertension and the importance of regular checkup and aggravating factors for the cases. The Helsinki Declaration was strictly followed for data collection. We conducted a face to face interview with a pretested structured questionnaire. To maintain the validity of the data collection tool, a structured questionnaire was developed and translated to local languages (Amharic and Afan Oromo) and back translated to English.

Powered by Editorial Manager® and ProduXion Manager® from Aries Systems Corporation

**Format for specific study types**<br><br>**Human Subject Research (involving human participants and/or tissue)**<br>• Give the name of the institutional review board or ethics committee that approved the study<br>• Include the approval number and/or a statement indicating approval of this research<br>• Indicate the form of consent obtained (written/oral) or the reason that consent was not obtained (e.g. the data were analyzed anonymously)<br><br>**Animal Research (involving vertebrate animals, embryos or tissues)**<br>• Provide the name of the Institutional Animal Care and Use Committee (IACUC) or other relevant ethics board that reviewed the study protocol, and indicate whether they approved this research or granted a formal waiver of ethical approval<br>• Include an approval number if one was obtained<br>• If the study involved *non-human primates*, add *additional details* about animal welfare and steps taken to ameliorate suffering<br>• If anesthesia, euthanasia, or any kind of animal sacrifice is part of the study, include briefly which substances and/or methods were applied<br><br>**Field Research**<br><br>Include the following details if this study involves the collection of plant, animal, or other materials from a natural setting:<br>• Field permit number<br>• Name of the institution or relevant body that granted permission	
**Data Availability**<br><br>Authors are required to make all data underlying the findings described fully available, without restriction, and from the time of publication. PLOS allows rare exceptions to address legal and ethical concerns. See the PLOS Data Policy and FAQ for detailed information.	Yes - all data are fully available without restriction

Powered by Editorial Manager® and ProduXion Manager® from Aries Systems Corporation

A Data Availability Statement describing where the data can be found is required at submission. Your answers to this question constitute the Data Availability Statement and **will be published in the article**, if accepted.<br><br>**Important:** Stating 'data available on request from the author' is not sufficient. If your data are only available upon request, select 'No' for the first question and explain your exceptional situation in the text box.<br><br>Do the authors confirm that all data underlying the findings described in their manuscript are fully available without restriction?	
**Describe where the data may be found in full sentences. If you are copying our sample text, replace any instances of XXX with the appropriate details.**<br><br>• If the data are **held or will be held in a public repository**, include URLs, accession numbers or DOIs. If this information will only be available after acceptance, indicate this by ticking the box below. For example: *All XXX files are available from the XXX database (accession number(s) XXX, XXX.).*<br>• If the data are all contained **within the manuscript and/or Supporting Information files**, enter the following: *All relevant data are within the manuscript and its Supporting Information files.*<br>• If neither of these applies but you are able to provide **details of access elsewhere**, with or without limitations, please do so. For example:<br><br>*Data cannot be shared publicly because of [XXX]. Data are available from the XXX Institutional Data Access / Ethics Committee (contact via XXX) for researchers who meet the criteria for access to confidential data.*<br><br>*The data underlying the results presented in the study are available from (include the name of the third party*	Data are available upon reasonable request where it is submitted to Mrs. Meron Hadis at merykiyaggg@gmail.com

and contact information or URL).<br>• This text is appropriate if the data are owned by a third party and authors do not have permission to share the data.<br><br>* typeset	
Additional data availability information:	Tick here if the URLs/accession numbers/DOIs will be available only after acceptance of the manuscript for publication so that we can ensure their inclusion before publication.

Powered by Editorial Manager® and ProduXion Manager® from Aries Systems Corporation

Manuscript
Click here to access/download;Manuscript;Final edited manuscript.docx

Heavy khat (Catha edulis) chewing and dyslipidemia as a cardiovascular risk factor among hypertensive patients in Jimma, Ethiopia: Unmatched case-control study

Meron Hadis Gebremedhin

Affiliations

School of Medicine, College of Health Sciences and Medicine, Wolaita Sodo University, Wolaita Sodo, Ethiopia

Corresponding author: merykiyaggg@gmail.com

Phone number: +2519-16-39-78-36

Abstract

Background: Hypertension is a state of high blood pressure; among the cardiovascular disease risk factors dyslipidemia and khat chewing were increasing at an alarming rate in Ethiopia but still neglected. This study aimed to assess heavy khat (Catha edulis) chewing and dyslipidemia as a cardiovascular risk factor among hypertensive patients in Southwest Ethiopia.

Methods: A facility-based case-control study was conducted on hypertensive and non-hypertensive individuals recruited from the chronic illness clinic of Jimma University medical center. ~~The~~ consecutive sampling technique was employed, and data were collected from May 15 to July 30, 2017. The data were entered using Epi data version 3.1 and exported to SPSS Version 21 for analysis. Logistic regression was performed, and statically significant were considered a cardiovascular risk factor among hypertensive patients.

Results: Of the 406 participants, 136 hypertensive cases and 270 non-hypertensive controls were involved. Most were TC/HDL ratio of >5 (72.1%) and (23.7%) of controls had dyslipidemia. Among Khat chewers, 54.67% had dyslipidemia; 72.9% use packed oils for food preparation of which 72.5% had dyslipidemia. Educational status (7.33; 2.66-20.19), excess alcohol consumption (3.33; 1.69-6.57), moderate khat chewers (3.54; 1.83-6.84), heavy khat chewers (19.12; 4.62-79.06), and vigorous work-related activity (3.84; 1.95-7.53), low HDL level (0.56; 0.32-0.97), dyslipidemia (11.83; 6.71-20.87) increases the odds of hypertension.

Conclusion: In this study, dyslipidemia and heavy khat chewing were statistically associated. ~~And both are the major risk factors.~~ Therefore, due consideration should be given to prevent and control risk factors by designing health information provision systems; promoting acceptable health practices. It should give particular attention to those growing farmers to minimize khat cultivation and awareness creation to the use of khat and increase educational attainment, change related behavioral factors, decrease work-related stress and focus on the type of oil.

Keywords: Dyslipidemia, Khat chewing, Hypertension, Case-control

Introduction

Hypertension is a high blood pressure defined in adults as systolic blood pressure greater than or equal to 140mmHg and/or diastolic blood pressure greater than or equal to 90mmHg. Rarely associated with understandable symptoms; it is a significant risk factor for cardiovascular disease and other vascular diseases, stroke, and/or chronic kidney disease (1). Africa as a whole, including the rural population, needs more consideration of cardiovascular health by responsible authorities (2). A normal blood pressure of cardiovascular benefit extends from lower systolic to lowest diastolic of 105mmHg to 60 mmHg. Globally, cardiovascular disease accounts for about 17 million deaths per year, accounting for one-third total death (3). From this, yearly around 9.4 million deaths are because of hypertension worldwide. Hypertension accounts for at least 45% and 55% mortality because of heart disease and stroke (4,5). Different pathogenesis of cardiovascular disease results from an abnormal increase in blood pressure and heart rate (6,7).

Khat (Qat, Kat and Miraa, Catha edulis) is a dicotyledonous evergreen flowering tree. It is a member of Celastraceae that grows in equatorial climates largely in the horn of Africa and the Arabian Peninsula (8). Ethiopia is one of the five main khat cultivating countries. Around 20 million people worldwide believed to be khat chewers, previously localized to East Africa and the Arabian peninsula (9). Chewing is the fundamental mechanism where it is the fresh leaves of khat chewed slowly for several hours. It contains over 40 compounds having the same effect **as** amphetamine and mainly glycosides, flavonoids, tannins, alkaloids, and sterols (7). Its consequence on the cardiovascular system is increasing heart rate, blood pressure, and vasomotor effects on the coronary vessels. The mechanism of action of khat is mainly the release of the neurotransmitter from the pre-synaptic vesicle (10). In Ethiopia, a study showed 37.9% of males and 9.2% of females chew khat (11). A study in Yemen shares the rise in BP to the duration of

khat chewing (12). But another study failed to associate khat as a risk factor for hypertension and other cardiovascular complications (13). Also, acute coronary vasospasm and myocardial infarction were other complications of high blood pressure (14). Chewing khat repeatedly and frequently will increase blood pressure and its difficulty of myocardial infarction (15,16). When we compare khat chewers to the non-chewers prevalence of hypertension and diastolic blood pressure is high among chewers (17). A recent report of WHO estimates, death because of CVD annually accounts for 17.7 million, and abnormal lipid profile results in a higher risk for CVD (18). From the different risk factors for the development of CVD, dyslipidemia is the most crucial factor. Mortality for CVD is high worldwide. Since the prevalence of hypertension and the growing prevalence of khat chewing in Africa, including our country, this study's findings should alarm the organizations working on public health issues (19).

There are several studies concerning khat's physiological and biochemical effects on humans. However, studies on specific heavy khat chewing and its association with dyslipidemia are not well-designed. This study picked up khat chewing and dyslipidemia as cardiovascular disease risk factors and the association of the two variables and other factors to the study area and our country, Ethiopia.

Methods and Materials

Study setting and period

The study was conducted from May 15 to July 30, 2017, at Jimma University medical center, Southwest Ethiopia. Jimma zone is located 600 km to the Southwest of Addis Ababa, Ethiopia's capital. JUMC is the only referral hospital in the Zone, providing services for approximately 9000 inpatients and 80,000 outpatient clients per year with a catchment population of about 15 million people. The Hospital health service includes outpatient departments, intensive care unit, Obstetrics and Gynecology, and inpatient services (medical, pediatrics, and surgical wards). The hospital also has medical, surgical, and gynecological referral clinics and an emergency intervention unit. The internal medicine department has about 2781 annual admission, and hypertension accounts for 13.2% (20).

Study design and population

A facility-based unmatched case-control study was employed. The source population was all adult patients (18 and above years of age) admitted and on a follow-up to the Jimma University medical center's medical wards. The study population was all hypertensive patients following at chronic illness clinic during the data collection period and those who fulfilled the eligibility criteria. Cases were hypertensive patients (whose systolic blood pressure is ≥140 mmHg and/or diastolic blood pressure ≥90 mmHg or those taking antihypertensive drugs during the study period). Controls were those residents in the study settings age above 18years old whose blood pressure is <140 mmHg/<90 mmHg during the study period and those who did not report a history of hypertension before and during the study period. I selected two controls for each case.

Inclusion criteria for cases were those patients diagnosed with hypertension (BP ≥ 140/90) and attended services during the study period selected consecutively from the follow-up clinic. Those newly diagnosed with high blood pressure linked to the follow-up clinic during the data collection period and reconfirmed by the re-measurement of blood pressure. Pregnant women, a client with renal disease, age less than 18 years old, mentally unstable or critically ill, and who could not respond excluded from the study.

I chose controls from the same medical centers that seek other services. These were patients from the same medical centers seeking other services that proved to be free from hypertension (diagnosed as a normal BP and confirm by the measurement of BP) during the data collection period. As exclusion, pregnant women, a client with renal disease, age <18 years old, mentally unstable or critically ill, were excluded from the study.

Sample size and sampling technique

Sample size

The standard sample size formula for unmatched case-control studies calculated the desired sample size. Using Epi info stat calc version3.1 with the following assumption; 95% confidence interval, 80% power, 2:1 control to case ratio and proportion of exposure among control groups = 20.9%, percentage of exposure among cases = 48.3% and Odds ratio:2.06 with 10% non-response rate the total sample size was 406 (136 cases and 270 controls).

Sampling technique

Consecutive sampling technique applied to recruit the required sample sizes of both groups (cases or controls). Selected study participants were interviewed upon their exit from the chronic illness clinic and to avoid overlapping we put a mark on the patient's card.

Data collection procedure

Data were collected using pre-tested interviewer-administered structured questionnaires, adapted with some modification from the WHO standard questionnaire (21) and previous studies (22–24). To check internal consistency, we translated the questionnaire into the local language (Oromia) and subsequently re-translated it back to English by different language experts. Doctors in internship facilitated data collection, six BSc nurses, and two ~~laboratories~~ with extensive experience in conducting data collection had collected the data. A structured questionnaire including socio-demographic parameters, behavioral and dietary risk factors was developed. And measurements such as weight, height, and three BP records included.

Arterial blood pressure: We measured blood pressure according to a standardized protocol with an automatic oscillometric method (dynamap).

The averages of systolic and diastolic blood pressure from the left and the right arm after 5 minutes of rest was used because per arm single measurement of blood pressure was available. We diagnosed hypertension as blood pressure >140/90 mmHg or patients with anti-hypertensive medications. As 6th Joint National Committee categorized hypertension as stage one and stage two, also it classified those patients taking the anti-hypertensive drug in stage two.

Normal: <120/<80

Pre-hypertension (high normal): 120-129/<80

Stage 1: 130-139/80-89

Stage 2: ≥140/≥90

Biochemical analysis

When the participants came in the morning after overnight fasting 5 ml of venous blood was collected; centrifuged and serum was used for biochemical tests. A lipid profile of the four parameters (TG, HDL, LDL, and TC) was done. Finally, we dichotomized the parameters and it is identified that there is a cardiovascular risk factor if there is dyslipidemia (where cholesterol to high-density lipoprotein ratio is high >5 or hypertriglyceridemia above 200-499mg/dl).

~~**Measurement variables**~~

The outcome variable was hypertension. The independent variables were socio-demographic factors such as educational, occupational, religious status, household income, and oral contraceptive use history. Non-modifiable determinants such as age, gender, family history of hypertension and modifiable lifestyle determinants such as smoking status, excess alcohol consumption, khat chewing, an anthropometric measurement like BMI, vigorous work-related activity and lipid profile (Total cholesterol, HDL, LDL, and triglyceride) were independent variables for this study.

~~**Operational definition**~~

~~Long time khat chewer had a history of chewing khat for over two years~~

~~Mild chewers: who chew less than one bundle of khat on one chewing occasion, moderate chewers who chew one bundle of khat, and heavy chewers chew above two bundles.~~

Since Ethiopian drinks like Tella, Areki, Teji, bordeare the main types of alcoholic drinks in the study area; alcohol consumption was defined as using these alcohols besides the standard ones.

In this study packed oil means packed in the jar "Chef, Hayat, Viking".

Vigorous work related activity is which makes you breathe much faster than an ordinary period.

Data processing and analysis

The data on each coded questionnaire was entered into Epi-data version 3.1 and double-entry verification was made and exported to SPSS version 21 statistical packages for analysis. The data were explored to check outliers, missing data, and assumptions. A chi-square test was used to assess each independent variable's association and the outcome variable. During analysis, frequencies of the different variables were determined as supposed necessary. Cross-tabulations and bivariate analysis were performed to select variables for multivariate analysis. Hence variables with a p-value < 0.25 in the bivariate analysis were taken as candidates for multivariable analysis. Finally, multivariable logistic regression analysis was performed to control possible confounding effects of variables. Variables with a p-value of less than 0.05 were taken statistically significant to identify risk factors and OR with its 95% CI was used to show the degree of association.

Data quality management

Data was pre tested in 5% of the total sample in another hospital rather than the study area and all required amendments were done based on the pre test. Experienced data collectors were required for data collection and supervision. A two- day intensive training was given for data collectors and supervisors about the aim of the study and sampling procedure. To minimize

measurement error anthropometric measurements were taken after prerequisites to avoid error i.e., Weight was measured after the scale pointer was checked at zero, subjects wear light clothes and stood straight and unassisted in the center of a balance platform. Height was also measured after participants were requested to remove their shoes, stood erect, a position at the plane with feet together and knee straight. The heels, buttock, shoulder blades were made straight against the stadiometer's vertical stand. Instrument calibration and random auditing were done and measurements were taken two times and finally, height to the nearest 0.1cm and weight to the nearest 0.1kg was taken. Selected participants were oriented about the study and their random selection; data from participants was correspondingly received in private settings after deep discussion that removed their doubts and cleared their confusion. Then subjects were interviewed measured for weight, height, and underwent blood pressure measurements and provided a blood sample. The same blood pressure recorder was used for the overall study population. Anthropometric measurements translated as per WHO Steps guidelines. Medical records and consultation with the person in charge of the patient were the gold standards for identifying the cases and controls.

Results

Socio-demographic characteristics of study participants

A total of 406 participants with a total of 136 hypertensive cases and 270 non-hypertensive controls were involved, making a response rate of 100%. More than half of cases 70(51.5%), and controls 143 (53.0%) were male participants. Nearly half 42.6% of both cases and controls were in the age group above >55 years age groups, and 20.6% of cases and 22.6% of controls were in the age group of <35 years being the median age being 51.56±14.92 years. More than half of the cases (60.3%) and (57.8%) of controls were living in urban areas, and concerning marital status, most of the cases (77.9%) and controls (80.4%) were married. One-fourth of the cases and nearly one-fourth of controls had no formal education. Approximately one-fourth of cases and 41% of controls had completed secondary education. More than half of the cases use oral contraceptives 65.2% and from the controls, 48.8% use OCP (Table 1).

Table 1: Socio-demographic characteristics of respondents

**Variable**		**Case No(%)**	**Control No(%)**	**Total No(%)**
Sex	Male	70(51.5%)	143(53.0%)	213(52.5%)
	Female	66(48.5%)	127(47.0%)	193(47.5%)
Age category	<35	28(20.6%)	61(22.6%)	89(21.9%)
	35-55	50(36.8%)	94(34.8%)	144(35.5%)
	>55	58(42.6%)	115(42.6%)	173(42.6%)
Place of residence	Urban	82(60.3%)	156(57.8%)	238(58.6%)

	Rural	54(39.7%)	114(42.2%)	168(41.4%)
Ethnicity	Oromo	96(70.6%)	184(68.1%)	280(69.0%)
	Amhara	17(12.5%)	34(12.6%)	51(12.6%)
	Other*	23(16.9%)	52(19.26%)	75(18.47%)
Marital status	Married	217(80.4%)	106(77.9%)	323(79.6%)
	Single/divorced/ widowed	53(19.6%)	30(22.1%)	83(20.4%)
Religion	Orthodox	29(21.3%)	77(28.5%)	106(26.1%)
	Muslim	87(64.0%)	156(57.8%)	243(59.9%)
	Other**	20(14.7%)	37(13.7%)	57(14.03%
Educational status	No formal education	34(25.0%)	61(22.6%)	95(23.4%)
	Primary education	78(57.4%)	113(41.9%)	191(47.0%)
	Secondary and above	24(17.6%)	96(35.6%)	120(29.6%)
Occupation	Farmer	48(35.3%)	74(27.4%)	122(30.0%)
	Government employee	25(18.4%)	49(18.1%)	74(18.2%)
	House wife	34(25.0%)	76(28.1%)	110(27.1%)
	Other***	29(21.3%)	71(26.3%)	100(24.6%)
Income	Low	78(57.4%)	132(48.9%)	210(51.7%)
	Middle	32(23.5%)	66(24.4%)	98(24.1%)
	High	26(19.1%)	72(26.7%)	98(24.1%)
OCP use	Yes	43(65.2%)	62(48.8%)	105(54.4%)
	No	23(34.8%)	65(51.2%)	88(45.6%)
Family hx of HPN	Yes	30(22.1%)	53(19.6%)	83(20.4%)

	No	106(77.9%)	217(80.4%)	323(79.6%)
Coffee consumption	Yes	124(91.2%)	229(84.8%)	353(86.9%)
	No	12(8.82%)	41(15.2%)	53(13.05%)

*Note: Other*Dawuro, Kafa, Yem** protestant, Catholic, Adventis***Student, merchant, Self-Employed, hx; history*

Biochemical factors

According to the American heart association, TC/HDL ratio should be <5. Among cases, most were a TC/HDL ratio of >5; 98 (72.1%) and 64 (23.7%) of controls had dyslipidemia. Almost half of the cases, 73 (53.7%) and 91 (33.7%) of the control group had a triglyceride level of >150mmg/dl. Among Khat chewers, 117 (54.67%) had dyslipidemia. Almost half (58.1%) of cases and 33.7% of controls have an abnormal total cholesterol level above 200mmg/dl, and triglyceride level was again a little higher among khat chewers 42.5% as compared to controls with 38%. Among khat chewers, 156(72.9%) use packed oils for food preparation, and from the 72.5% had dyslipidemia (Table 2).

Behavioural risk factors of hypertension

About 70.6% of cases and 43.7% of controls chewed khat in their lifetime. Most of the cases (43.4%) chew khat daily. Almost half 53.1% of cases chew one bundle of khat during one chewing occasion while from controls 66.9% chew less than one bundle. Most of the reason for chewing is religious prayer, increased social interaction, addiction, and staying awake.

More cases (56.6%) than controls (26.3%) drank alcohol. Among alcohol users, 35.8% of cases and 17.4% of controls drank “Tella”, “Tejj”, “areki” and beer three times a week. More than half (57.3%) cases and 63.0% of controls drank alcohol for more than 10 years. Duration of alcohol

drinking 10±4.41 minimum of 2 and maximum is 30. More cases (91.2%) than controls (84.8%) drank over 2 cups of coffee per day.

Only 38(27.9%) cases and 40(14.8%) controls had a previous history of smoking cigarettes but only 10.5% of cases and 2.5% controls were smokers during the time of data collection. About 44.7% of cases smoke daily and 30.6% of controls smoke daily. Most of the cases and controls 85.3% of cases and 84.8% of controls have no family members who smoke a cigarette. Number of cigarettes smoke per each day of smoking 4±3.01 minimum 1 and maximum 20. Moreover, 93(68.4%) of cases and 102(37.8%) of controls had vigorous work-related activity (Table 2).

Dietary risk factor

More controls (24.4%) ate fruit occasionally (at least once per week) compared with cases (9.8%). Most of cases (88.5%) and controls (80.9%) used packed oil for cooking. Concerning salt usage, 28.7% of cases use too much salt in their diet and among controls, 37% use a too little salt in their diet. Regarding anthropometric measurement, most of the study participants had normal body mass index range (18.5-24.9) (Table 2).

Table 2: Bivariate logistic regression analysis showing factors associated with as CVD risk factors

**Variable**	**Category**	**Case No (%)**	**Control No (%)**	**COR(95%, CI)**	**p-value**
**Educational Status**	No formal	34(25.0%)	61(22.3)	2.23(1.21-4.12)	0.01
	Primary	78(57.4%)	113(41.9%)	2.76(1.62-	<0.001

				4.70)	
	Secondary	24(17.6%)	96(35.6%)	1	
**Income(1birr=40dollar)**	Low	78(57.4%)	132(48.9%)	1.64(0.96-2.78)	0.068
	Middle	32(23.5%)	66(24.4%)	1.34(0.73-2.49)	0.35
	High	26(19.1%)	72(26.7%)	1	
**OCP use**	Yes	43(65.2%)	62(48.8%)	1.96(1.06-3.62)	0.032
	No	23(34.8%)	65(51.2%)	1	
**Hx of smoking**	Yes	38(27.9%)	40(14.8%)	2.23(1.35-3.69)	0.002
	No	98(72.1%)	230(85.2%)	1	
**Current smoking status**	Still smoking	4(10.5%)	1(2.5%)	4.83(0.51-46.18)	0.17
	Reduced	10(26.3%)	7(17.5%)	2.01(0.64-6.35)	0.23
	Ceased	24(63.2%)	32(80.0%)	1	
**Alcohol**	Yes	77(56.6%)	71(26.3%)	3.66(2.37-5.65)	<0.001
	No	59(43.4%)	199(73.7%)	1	
**Current alcohol hx**	Still drinking	14(18.2%)	27(38.0%)	0.33(0.15-0.72)	0.006

	Reduced	17(22.1%)	15(21.1%)	0.77(0.33-1.79)	0.54
	Ceased	46(59.7%)	29(40.8%)	1	
**Chew Khat daily**	Yes	96(70.6%)	118(43.7%)	3.09(1.99-4.80)	<0.001
	No	40(29.4%)	152(56.3%)	1	
**No of bundle**	<1 bundle	29(30.2%)	79(66.9%)	1	
	1 bundle	51(53.1%)	36(30.5%)	3.82(2.11-6.91)	<0.001
	>2bundle	16(16.7%)	3(2.5%)	14.01(3.84-51.79)	<0.001
**Physical activity**	Yes	93(68.4%)	102(37.8%)	3.56(2.30-5.52)	<0.001
	No	43(31.6%)	168(62.2%)	1	
**Type of oil**	Liquid	31(22.8%)	98(36.3%)	1	
	Solid	105(77.2%)	172(63.7%)	2.10(1.30-3.39)	0.002
**Salt consume**	Far too little	13(9.6%)	26(9.6%)	1	
	Too little	32(23.5%)	100(37.0%)	0.66(0.32-1.38)	0.28
	Just the right amount	35(25.7%)	75(27.8%)	1.77(0.82-3.82)	0.15
	Too much	39(28.7%)	45(16.7%)	159(0.73-3.44)	0.24

	Far too much	17(12.5%)	24(8.9%)	1.42(0.58-3.45)	0.44
**Important salt**	Very important	84(61.8%)	156(57.8%)	0.38(0.14-1.03)	0.06
	Somewhat important	32(23.5%)	101(37.4%)	0.22(0.08-0.63)	0.005
	Not important	10(7.4%)	6(2.2%)	1.17(0.29-4.73)	0.83
	I don't know	10(7.4%)	7(2.6%)	1	
**TC**	>200	79(58.1%)	91(33.7%)	2.73(1.78-4.17)	<0.001
	<200	57(41.9%)	179(66.3%)	1	
**HDL**	>40	119(44.1%)	77(56.6%)	1	
	<40	59(43.4%)	151(55.9%)	1.66(1.09-2.51)	0.017
**TG**	<150	63(46.3%)	179(66.3%)	1	
	>150	73(53.7%)	91(33.7%)	2.28(1.49-3.7)	<0.001
**LDL**	<100	33(24.3%)	123(45.6%)	1	
	>100	103(75.7%)	147(54.4%)	2.61(1.65-4.14)	<0.001
**TC/HDL**	<5	38(27.9%)	206(76.3%)	1	
	>5	98(72.1%)	64(23.7%)	8.26(5.17-13.19)	<0.001

**BMI**	<18.5	37(27.2%)	48(17.8%)	2.51(0.76-8.32)	0.13
	18.5-24.9	78(57.4%)	158(58.5%)	1.06(0.51-5.08)	0.42
	25-29.9	17(12.5%)	51(18.9%)	1.08(0.31-3.77)	0.90
	>30	4(2.9%)	13(4.8%)	1	

Note: Hx: history

Cardiovascular risk factors

Bivariate unconditional logistic regression was carried to check the association between dependent and independent variables. There was no difference between cases and controls among variables; sex, residence, ethnicity, occupation, family history of hypertension, BMI.

Variables associated with the outcome variable in the bivariate analysis (P ≤0.25) were taken to the multivariable analysis for further analysis. This is basically to compensate for the power of the test since negative findings (that is, p > 0.05) maybe just because of inadequate power. Those who chew khat daily have increased odds of having the disease as compared to controls by three times. But after adjusting for possible confounding factors in the analysis of unconditional logistic regression educational status, excess alcohol consumption, number of bundles chewed, vigorous work-related activity, low HDL, total cholesterol to /HDL ratio (dyslipidemia) were found to be independent predictors of hypertension.

Having no formal education increases the odds of developing hypertension by 7.33 times (AOR=7.33; 2.66-20.19) compared to controls with primary education and secondary and above educational status. Similarly, patients with a history of excess alcohol consumption had three times (AOR = 3.33; 1.69-6.57) higher odds of developing hypertension than those with no history of alcohol consumption. Considering the mean estimated quantity of khat chewed per session mild chewers (<1 bundle) were not shown to be at risk, while moderate khat chewers (1 bundle during one chewing session) were shown to be at high risk (OR = 3.82) and heavy khat chewers at even higher risk (OR = 14.01).

The odds of having the disease in those with vigorous work-related activity were three times as compared to the control groups (AOR=3.84; 1.95, 7.53). Also, having a low HDL level and

having total cholesterol to HDL ratio of five were independent risk factors for hypertension (Table 3).

The effect of age, family history of hypertension, consumption of vegetables, and drinking coffee disappeared in the multivariable analysis when adjusted for confounders.

Table 3: Multivariate logistic regression analysis showing factors associated as CVD risk factors in hypertension development

Variable	Category	AOR(95%, CI)	p-value	COR(95%, CI)	p-value
**Educational Status**	No formal	7.33(2.66-20.19)	<0.001	2.23(1.21-4.12)	0.01
	Primary	3.29(1.39-7.73)	0.006	2.76(1.62-4.70)	<0.001
	Secondary	1		1	
**Excess alcohol consumption**	Yes	3.33(1.69-6.57)	0.001	3.66(2.37-5.65)	<0.001
	No	1		1	
**No of bundle of khat chewed**	<1 bundle	1		1	
	1 bundle	3.54(1.83-6.84)	<0.001	3.82(2.11-6.91)	<0.001
	>2bundle	19.12(4.62-79.06)	<0.001	14.01(3.84-51.79)	<0.001
**Vigorous work related activity**	Yes	3.84(1.95-7.53)	<0.001	3.56(2.30-5.52)	<0.001
	No	1		1	
**Low HDL level**	>40	1		1	
	<40	0.56(0.32-0.97)	0.04	1.66(1.09-2.51)	0.017
**Dyslipidemia**	<5	1		1	
	>5	11.83(6.71-20.87)	<0.001	8.26(5.17-13.19)	<0.001

Discussion

Khat chewing is a common habit in the study area for different reasons, like increasing social interaction, religious ceremonies, increasing excitement and boosting memory with its easy availability in the local area. Current studies prove that effect on the increment of blood pressure (18). In this study, the fascinating result is that there is an association between dyslipidemia and khat. The study aimed to decide khat and dyslipidemia as a cardiovascular risk factor among hypertensive patients in Jimma, Southwest Ethiopia. This study found educational status, excess alcohol consumption, the number of bundles of khat chewed; vigorous work-related activity, and low HDL level and TC/HDL were cardiovascular risk factors in hypertensive patients.

Educational status affects the development of hypertension. Those who had no formal education were 7.33 times and those with primary education were 3.29 times more likely to be exposed to hypertension. This is in line with a study in Gaza Governorates where it is 5 times higher among low educational level ones with an adjusted odds ratio of(AOR=5.93, 95% CI, 3.0–11.82) (25). This is in contrast to a study in Bale, Nekemte Ethiopia, and Korea were having secondary and above educational level increases the chance of development of hypertension (23,26,27). These might be because of the socioeconomic status study population, cultural, dietary and lifestyle differences of the areas and countries.

Moderate khat chewers (1 bundle during one chewing session) and heavy khat chewers (>2 bundles during one chewing session) had a higher risk to develop a disease. This is in line with a study in Bahirdar, Ethiopia that associates khat chewing with elevated systolic blood pressure (28). A study in Butajira, Ethiopia also showed regular khat chewing will increase diastolic blood pressure (17). Another observational study in Yemeni khat chewers who had acute

coronary syndrome showed that khat chewers had worse outcomes than non-chewers (9). But in contrast, another study in Ethiopia found there is no association (23). This may support the fact that khat chewing is a powerful cardiovascular determinant and reducing or cessation is the single most effective lifestyle measure for the prevention of a large number of deaths and disabilities related to hypertension. And for the difference, there needs to be a more experimental study should be carried on in the future.

In this study, community obesity is not a problem; there is no association between anthropometric measurements and being hypertensive. This might be due to the high consumption of khat in the study population as well as the community. Because khat chewing results in anorexia, which suppresses appetite (7). But to surprise, dyslipidemia and level of HDL are low, showing that khat has affects the development of abnormal lipid profiles. In this study, it was found that most khat chewers had dyslipidemia and a low level of HDL. Thus, khat consumption affects the development of hypertension directly and indirectly by increasing risk factors like dyslipidemia (18).

Several researchers reported sedentary lifestyle or physical inactivity is related to hypertension but this study found participants who engaged in vigorous work-related activities were more likely to be hypertensive as compared to those who engaged in moderate work-related activities. This study is in line with a study in Tigray, north Ethiopia (24). This might be because stressful activities increase stress in everyday life activity and release of stress hormones. But to compare those who have a sedentary lifestyle, moderate physical activity is protective but different studies were against this (19,28). Excess alcohol consumption increases hypertension three-fold because of its sympathetic effect and in line with other studies (30–32).

It is the first study to provide an association between dyslipidemia and hypertension in the study area. I had selected institution-based cases to minimize selection bias, and this study used a strong study design and incorporated lipid profile as one risk factor. However, recall bias might affect the study's result as the previous history was asked and confounders effect because of unmatched selection of study subjects. In this study novel, risk factors were not included because of the absence of laboratory equipment and reagent in the study area.

Conclusion

The study showed there is a high incidence of dyslipidemia and heavy khat chewing in the study area. Dyslipidemia, manifested as Low HDL level and high total cholesterol to HDL ratio, is a sign of cardiovascular risk factor. Education status, vigorous work-related activity, excess alcohol consumption, and moderate to heavy khat chewing were the main cardiovascular risk factors in hypertensive patients. In the study area, khat chewing was a common habit, so it needs special attention to decrease this habit.

Ethical approval and consent to participants

Jimma University institute of health science and the ethical review committee approved the study; the department of biomedical science and internal medicine provided a support letter. All the study participants were informed about the purpose of the study, their right to refuse, and assured confidentiality and verbal and written consent was obtained before the interview. At the end of each interview and measurement procedure, we made awareness creation to study participants in control groups about cardiovascular disease and hypertension's risk factors and the importance of regular checkups and aggravating factors for the cases. We conducted a face-to-face interview with a pretested structured questionnaire. To maintain validity of the data

collection tool's, a structured questionnaire was developed and translated to local languages (Amharic and Afan Oromo) and back-translated to English.

Patient consent for publication: Not required

Competing interests

None declared

Data availability statement: Data are available upon reasonable request where it is submitted to Mrs. Meron Hadis at merykiyaggg@gmail.com

Funding

No funding received but Jimma University paid per diam for data collectors

Authors' contributions

MH: Conceived the study, involved proposal development, supervision, and data analysis and manuscript preparation and guided overall stage of the study.

Author Information

MH: MSc, School of medicine, Wolaita Sodo University, Ethiopia.

Acknowledgement

I thank Jimma University and thank with the deepest gratitude all participants who took part in this study, my families and friends who were with me and illuminated me with their presence.

List of Abbreviations

AOR: Adjusted odds ratio

BMI: Body mass index

COR: Crudes odds ratio

CVD: Cardiovascular disorder

HDL: High density lipoprotein

LDL: Low density lipoprotein

TC: Total cholesterol

TG: Triglycerides

WHO: World health organization

Reference

1. Kearney PM, Whelton M, Reynolds K, Muntner P, Whelton PK, He J. Global burden of hypertension: analysis of worldwide data. Lancet. 2005;365(9455):217–23.

2. Bosu WK, Reilly ST, Aheto JMK, Zucchelli E. Hypertension in older adults in Africa: A systematic review and meta-analysis. PLOSEONE. 2019;14(4):1–25.

3. WHO. A global brief on Hypertension World Health Day 2013. 2013;

4. Haldar RN. Global Brief on Hypertension: Silent Killer, Global Public Health Crisis. Indian J Phys Med Rehabil. 2013;24(1):2–2.

5. Suhendra AD, Asworowati RD, Ismawati T. World health statistics, 2020 [Internet]. Vol. 5, Akrab Juara. 2020. 43–54 p. Available from: http://www.akrabjuara.com/index.php/akrabjuara/article/view/919

6. Gebrie A, Alebel A, Zegeye A, Tesfaye B. Prevalence and predictors of khat chewing among Ethiopian university students : A systematic review and meta-analysis. PLoS One. 2018;76:1–15.

7. Wabe NT. Chemistry , Pharmacology , and Toxicology of Khat (Catha Edulis Forsk): A Review. Addict Heal Summer Autumn; 3(3):137–49.

8. Al-Motarreb A, Baker K, Broadley KJ. Khat: Pharmacological and medical aspects and its social use in Yemen. Phyther Res. 2002;16(5):403–13.

9. Ali WM, Habib KF Al, Al-motarreb A, Singh R, Hersi A, Faleh H Al, et al. Acute Coronary Syndrome and Khat Herbal. 2011;

10. Patel NB. Khat (Catha edulis Forsk) – And now there are three. Brain Res Bull [Internet]. 2019;145(May 2018):92–6. Available from: https://doi.org/10.1016/j.brainresbull.2018.07.014

11. Abate A, Tareke M, Tirfie M, Semachew A, Amare D, Ayalew E. Chewing khat and risky sexual behavior among residents of Bahir Dar City administration , Northwest Ethiopia. Ann Gen Psychiatry [Internet]. 2018;1–9. Available from: https://doi.org/10.1186/s12991-018-0194-2

12. Toennes SW, Harder S, Schramm M, Niess C, Kauert GF. Pharmacokinetics of cathinone , cathine and norephedrine after the chewing of khat leaves. 2003;125–30.

13. Mega TA, Dabe NE. Khat (Catha Edulis) as a Risk Factor for Cardiovascular Disorders : Systematic Review and Meta-Analysis. Open Cardiovasc Med J. 2017;11:146–55.

14. Hassen K, Abdulahi M, Dejene T, Wolde M, Sudhakar M. Khat as a risk factor for hypertension: A systematic review. JBI Evid Synth [Internet]. 2012;10(44). Available from: https://journals.lww.com/jbisrir/Fulltext/2012/10440/Khat_as_a_risk_factor_for_hypertension__A.1.aspx

15. Ayano G, Yohannis K, Abraha M. Epidemiology of khat (Catha edulis) consumption among university students: A meta-analysis. BMC Public Health. 2019;19(1):1–13.

16. Tesfaye F, Byass P, Berhane Y, Bonita R, Wall S. Association of smoking and khat (Catha edulis Forsk) use with high blood pressure among adults in Addis Ababa, Ethiopia, 2006. Prev Chronic Dis. 2008;5(3).

17. Getahun W, Gedif T, Tesfaye F. Regular Khat (Catha edulis) chewing is associated with elevated diastolic blood pressure among adults in Butajira, Ethiopia: A comparative study. BMC Public Health. 2010;10:4–11.

18. Al-Duais MA, Al-Awthan YS. Association between qat chewing and dyslipidaemia among young males. J Taibah Univ Med Sci [Internet]. 2019;14(6):538–46. Available from: https://doi.org/10.1016/j.jtumed.2019.09.008

19. Mega TA, Dabe NE. Khat (Catha Edulis) as a Risk Factor for Cardiovascular Disorders : Systematic Review and Meta-Analysis. Cardiovasc Med J. 2017;11:146–55.

20. Press D. Prevalence of hypertension and its risk factors in southwest Ethiopia : a hospital-based cross-sectional survey. Dovepress. 2013;(February 2015).

21. WHO. WHO STEPS Instrument For non-communicable disease.

22. Abdul-razak S, Daher AM, Ramli AS, Ariffin F, Mazapuspavina Y, Ambigga KS, et al. Prevalence , awareness , treatment , control and socio demographic determinants of hypertension in Malaysian adults. BMC Public Health [Internet]. 2016;1–10. Available from: http://dx.doi.org/10.1186/s12889-016-3008-y

23. Study C, Minda Z, Lencha B, Wordofa D, Lemessa F. Determinants of Hypertension Among Patients in Bale Zone , Southeast. Acute chronic Dis reoprts. 2018;2(1):1–9.

24. Bekele A. Determinants of Hypertension among Adults in Tigray , North Ethiopia : A Matched Case Control Study. Int J Noncommunicable Dis. 2018;2(January).

25. Abed Y, Abu-Haddaf S. Risk Factors of Hypertension at UNRWA Primary Health Care Centers in Gaza Governorates. ISRN Epidemiol. 2013;2013:1–9.

26. Geleta GT, Cheme MC, Roro EM. Physical , behavioral and sociodemographic determinants of hypertension among the adult population in Nekemte town , western Ethiopia : community based study. BMC Res Notes [Internet]. 2019;4–9. Available from: https://doi.org/10.1186/s13104-019-4804-0

27. Baek T, Lee H, Lim N, Park H. Gender differences in the association between socioeconomic status and hypertension incidence : the Korean Genome and Epidemiology Study (KoGES). BMC Public Health [Internet]. 2015;1–7. Available from: http://dx.doi.org/10.1186/s12889-015-2175-6

28. Birhane BW. The Effect of Khat (Catha edulis) Chewing on Blood Pressure among Male Adult Chewers, Bahir Dar, North West Ethiopia. Sci J Public Heal. 2014;2(5):461.

29. Mulugeta Y, Id D, Firew BS. Factors associated with noncommunicable disease among adults in Mecha district , Ethiopia : A case control study. PLOSEONE. 2019;1–13.

30. Government US. Alcohol consumption, obesity and hypertension: Relationship patterns along different age groups in Uganda. 2019;

31. Geta TG, Woldeamanuel GG, Hailemariam BZ, Bedada DT. Association of chronic khat chewing with blood pressure and predictors of hypertension among adults in gurage zone, southern Ethiopia: A comparative study. Integr Blood Press Control. 2019;12:33–42.

32. Santana NMT, Mill JG, Velasquez-Melendez G, Moreira AD, Barreto SM, Viana MC, et al. Consumption of alcohol and blood pressure: Results of the ELSA-Brasil study. PLoS One. 2018;13(1):1–13.

Click here to access/download
Supporting Information
Commented Manuscript.docx
