# Supplementary material for: Heavy khat (Catha edulis) chewing and dyslipidemia as modifiable hypertensive risk factors among patients in Southwest, Ethiopia: Unmatched case-control study
Source: PLoS One. 2021 Oct 26;16(10):e0259078. doi: 10.1371/journal.pone.0259078 (PMC8547649; doi:10.1371/journal.pone.0259078)
Supplement: Attachment — Submitted filename: response to reviewers.docx [file pone.0259078.s006.docx]

**Manuscript Title:** Heavy khat (Catha edulis) chewing and dyslipidemia as modifiable hypertensive risk factors among patients in Southwest, Ethiopia: Unmatched case-control study

**Dear editor and reviewers**

We would like to extend our heartfelt gratitude and appreciation for your valuable comments and priceless time. Thank you, your comments have helped us a lot to improve the manuscript and focus on important points and also a base for our future performance. We tried to address all comments point by point in this paper.

# Responses for the editor

1. Please ensure that your manuscript meets PLOS ONE's style requirements, including those for file naming.

**Response:** Comment accepted and the manuscript has been revised accordingly to meet PLOS ONE's style requirements including naming of tables.

2. Please include additional information regarding the questionnaire used in the study and ensure that you have provided sufficient details that others could replicate the analyses. For instance, if you developed a questionnaire as part of this study and it is not under a copyright more restrictive than CC-BY, please include a copy, in both the original language and English, as Supporting Information. Moreover, please include more details on how the questionnaire was pre-tested, and whether it was validated.

**Response:** Comment accepted and changes have been made, please see the highlighted manuscript from line (146-162). And the tool we used for this study was submitted as supplementary material both in English and Amharic language.

The questionnaire was validated in the following way. After the initial pool of questionnaire items were written, qualified experts reviewed the items to make sure they are accurate, free of item construction problems, and grammatically correct. Before the study was conducted in the intended participants pretest was conducted on 5% of sample size other than the study hospital to clear confusion about any items, and whether respondents have suggestions for possible improvements of the items. We analysed responses to the pilot and feedback from our interviewers on interviewees’ level of understanding and any questions that caused interviewee confusion or difficulty. We then used this information to revise the questionnaire so as to further ensure clarity and comprehensibility.

3. We note that you have indicated that data from this study are available upon request. PLOS only allows data to be available upon request if there are legal or ethical restrictions on sharing data publicly.

**Response:** Comment accepted and amendment was done. The data sets used and/or analyzed during this study are available anytime from the corresponding author.

4. We note that the grant information you provided in the ‘Funding Information’ and ‘Financial Disclosure’ sections do not match.

**Response:** Comment accepted and amendment has been done. We need the funding statement to be read as “Jimma University covered perdium for data collectors and supervisors” and this funding statement was included in the cover letter.

5. Your ethics statement should only appear in the Methods section of your manuscript. If your ethics statement is written in any section besides the Methods, please move it to the Methods section and delete it from any other section. Please ensure that your ethics statement is included in your manuscript, as the ethics statement entered into the online submission form will not be published alongside your manuscript.

**Response:** Comment accepted and amendment has been done, please see the highlighted manuscript from line (213-218).

# Response to reviewer 1

1. The manuscript needs to be extensively revised for language (grammar and mechanics) starting from the title. As it currently stands, it is unsuitable for publication.

**Response:** Comment accepted and changes have been made to the whole document.

2. The study is not about cardiovascular risk factors among hypertensive patients. It is about risk factors for hypertension. Hence, the title needs to be accordingly revised.

**Response:** Comment accepted and changes have been made. Please see the highlighted manuscript from line (1-3).

3. Make sure that the findings presented in the results part of the abstract make sense. For example, is the finding that work-related vigorous physical activity increases the odds of hypertension plausible and logical? The author needs to re-check the analysis. Also, to what level of education does “educational status” refer to? The author should also accompany abbreviations with their expanded form when they first appear in the abstract.

**Response:** Comment accepted and amendments have been done. Please see the highlighted manuscript from line (27-34).

4. Furthermore, the author should conclude only based on the findings. This study has nothing about whether minimizing khat cultivation by farmers would have an effect in reducing khat consumption and thereby the risk of hypertension. In countries where khat is not cultivated, it may be imported from other countries. Reducing cultivation may not necessarily reduce khat consumption. That conclusion is off-topic. The recommendation about increasing educational attainment also does not seem to be feasible and may not have an effect in the short term. The recommendations about stress and type of food oil are also not supported by the findings of the present study.

**Response:** Very sorry for the deviation out of the objective thanks for your valuable comment. Comment accepted and changes have been made. Please see the highlighted manuscript from line (35-40).

5. The arguments in the introduction lack coherence. Make sure that you coherently address one main idea in a paragraph. As the introduction currently stands, each paragraph seems to entertain multiple unrelated ideas and hence makes reading through it difficult. Accompany all abbreviations with their full form when they appear first in the body of the manuscript.

**Response:** Comment accepted and changes have been made. Please see the highlighted manuscript from line (45-82).

6. In the last paragraph of the introduction, the argument about the objectives of the present study is confusing. Is the aim of the study to investigate the association between khat chewing and dyslipidemia or the association of khat chewing and dyslipidemia with hypertension? You should clearly re-write this argument.

**Response:** Comment accepted and modifications have been made. Please see the highlighted manuscript from line (83-89).

7. Reference #20 does not seem a proper reference for the information presented about the study setting. For one thing, it is out-of-date. Besides, it is not an original source for such information.

**Response:** Comment accepted and changes have been made. Please see the highlighted manuscript from line (94-99).

8. Description of the study population should be revised. It doesn't sound right. On the one hand, all hypertensive patients do not seem to be in the study population. They were recruited through a sampling procedure based on a sample size calculated a-priori. On the other hand, the study is not limited solely to hypertensive patients.

**Response:** Comment accepted and changes have been made. Please see the highlighted manuscript from line (101-112).

9. While defining cases on page 5, the author should clarify based on how many occasions of blood pressure measurement hypertension was defined.

**Response:** Thanks for your comment. For case selection, as cases were those already diagnosed for being hypertensive and also who were taking anti-hypertensive drugs. In the selection of controls hypertension was confirmed by three consecutive measurements of blood pressure. Please see the highlighted manuscript from line (106-110).

10. When describing the methodology, do not do it in the first person singular. Eg., "I selected two controls for each case." (Page 5, last line.)

**Response:** Comment accepted and amendments have been done.

11. Description of the inclusion criteria for both the cases and the controls on page 6 must be properly revised. For one thing, the description is unnecessarily long. Besides, the criteria should be clear and properly justified. For example, once you stated that the population studied was comprised of adults > 18 years, then no need of stating that persons under 18 years were excluded.

**Response:** Comment accepted and changes have been made. Please see the highlighted manuscript from line (106-112).

12. The description of the sample size calculation on page 6 is unclear. The language is awkward and the assumptions are not clearly stated. For example, what was the exposure variable? You also need to cite reference for the assumptions about the magnitude of exposure among the cases and the controls. Further, what do you mean by the "standard sample size formula"? If you used software to calculate the sample size, better to omit the description about the formula.

**Response:** Comment accepted and modifications have been made. Please see the highlighted manuscript from line (137-140). The exposure variable was past history of cigarette smoking from southeast, Ethiopia.

13. Merge the description of the sampling procedure with the description of the sample size. Further, the description of the sampling procedure is too brief and too vague. Clear and sufficient description of the sampling procedure both for the cases and the controls should be provided.

**Response:** Comment accepted and amendment has been done. Please see the highlighted manuscript from line (137-144).

14. In the data collection section (page 7), write the proper name of the local language - Afaan Oromoo.

**Response:** Comment accepted and changes have been made. And we included the questionnaire translated in to Amharic as supporting information.

15. Besides, the description about the definition of hypertension is unclear. Also the description of hypertensive patients on medication is confusing. Should be re-written.

**Response:** Thanks for your comment. Hypertension was diagnosed based the criteria of American College of cardiology classification and those patients already diagnosed and confirmed of having hypertension and started anti-hypertensive drugs were categorized as stage 2.

16. Remove the operational definitions section (pages 8 & 9) and provide a description of how the different variables were measured and operationalized under the variables of the study section (page 8).

**Response:** Comment accepted and amendment has been done. Please see the highlighted manuscript from line (114-135).

17. Further, the main independent variables, namely heavy khat use and dyslipidemia need explicit elaboration. As the description stands now, it is difficult to properly understand how the IDVs were measured and operationalized. As necessary, sources for the given definitions need to be provided by way of reference citation.

**Response:** Comment accepted and amendment has been done. Please see the highlighted manuscript from line (123-135).

18. Assimilate the data quality management section into the data collection procedure section.

**Response:** Comment accepted and amendment has been done. Please see the highlighted manuscript from line (146-188).

19. Under the socio-demographic characteristics section, describe only the salient socio-demographic features in text. The rest can be seen from the table. Also clearly indicate in terms of which characteristics the cases and controls significantly differ.

**Response:** Comment accepted and amendment has been done. Please see the highlighted manuscript from line (222-227).

20. In Table 1, do a statistical comparison of the cases and controls to show whether or not they were balanced in terms of basic characteristics. You may use chi-square test.

**Response:** Comment accepted and changes have been made. Please see Table.

21. The descriptive results about the biochemical, behavioural and nutritional characteristics of the study participants (pages 13-14) are unclear and confusing. Just provide a brief description of the salient aspects of these findings under one sub-heading titled "biochemical, behavioural and nutritional characteristics". Like in Table 1, here also provide in a table the statistical comparison of the cases and the controls in terms of characteristics provided. Replace Table 2 with a table similar to the one I suggested for Table 1.

**Response:** Comment accepted and change has been made. Please see the highlighted manuscript from line (229-248).

22. The study is specifically about risk factors for hypertension. Hence you should modify the sub-heading "Cardiovascular risk factors" on page 19 with a more descriptive sub-heading such as "Risk factors for hypertension".

**Response:** Comment accepted and changes have been made. Please see the highlighted manuscript line 249.

23. Avoid discussion from the results section. For example, the arguments about choice of analytical methods provided on page 19 may be justified in the methods section, not in the results. Further, such arguments must be substantiated by appropriate reference citations to convince readers about the trustworthiness of the claims.

**Response:** Comment accepted and amendment has been done. Please see the highlighted manuscript from line (205-208).

24. In the methods section, nothing was described about the use of unconditional logistic regression but in the results (page 19) there is a claim of the use of such method. If unconditional logistic regression was used, then in the methods section the use of this method should be clearly described.

**Response:** Comment accepted and change has be made. There was a typing error.

25. As heavy khat use has not been clearly defined in the methods section, it is difficult to understand the result pertaining to the association of heavy khat use and hypertension on page 19.

**Response:** Heavy khat chewers where those who had a history of khat chewing of two or more bundles of khat in one chewing session, moderate chewers were those who chew almost a bundle of khat and those mild chewers were those who chew less than one bundle of khat for one chewing session at least for more than six months.

26. Further, as already commented in the abstract, the finding that vigorous work-related activity increases the odds of hypertension is not plausible. The authors should re-do the analysis using an appropriate reference category and check the findings.

**Response:** Comment accepted and changes have been made. Please see the highlighted manuscript line (267-269). There was a problem on selecting the reference group and a different variable was used for analysis, great excuse.

27. In Table 3, the columns for AOR and COR should be transposed. Also better to omit the p-value columns.

**Response:** Comment accepted and changes have been made. Please see Table 3 analysis and categorization has been done again.

28. In Table 3, is the number of bundles chewed per day or per session or per what?

**Response:** Thanks for the comment. It is per one chewing session.

29. The author should revise the discussion to make it more mature and scholarly.

**Response:** Comment accepted and changes have been made. Please see the edited discussion.

30. Remove the introductory statement about khat from the first paragraph of the discussion. In the first paragraph, just summarize the main findings and discuss those findings in subsequent paragraphs.

**Response:** Comment accepted and changes have been made. Please see the highlighted manuscript line (276-280).

31. Do not sensationalize the discussion of the findings (e.g., “But to surprise”, line 310, page 23).

**Response:** Comment accepted and changes have been made.

32. Your study did not investigate the association of khat chewing with dyslipidemia and HDL. Your argument on page 23 (line 310-311) is not supported by your data and is misleading.

**Response:** Comment accepted and changes have been made. Please see the highlighted manuscript line(290-303).

33. Further, the result pertaining to the association between dyslipidemia and hypertension needs to be sufficiently discussed.

**Response:** Comment accepted and changes have been made. Please see the highlighted manuscript line (322-330).

34. As already commented above, the claim on the last paragraph of page 23 that work-related vigorous physical activity increases the odds of hypertension is counterfactual and not plausible. You need to re-check your analysis and update the discussion accordingly.

**Response:** Comment accepted and changes have been made. Please see the highlighted manuscript line (304-310).

35. The first sentence in the last paragraph of the discussion (page 24) that the present study is the first study in the study setting seems an exaggeration of the findings. There may already be a study which the author failed to find. At least, it is already known that dyslipidemia is a risk factor for hypertension. Revise or avoid such claim.

**Response:** Comment accepted and changes have been made.

36. Clearly discuss the limitations of this study.

**Response:** Comment accepted and changes have been made. Please see the highlighted manuscript line (332-334).

37. The conclusion seems to stray away from the objective of the study and from what the data shows. For example, the first sentence of the conclusion section is wrong and not in line with the objective of the study. The second sentence is also unclear and not in line with the study's objective. Generally, the author should revise the conclusion to be in line with the objective of the study and suggest a pertinent recommendation about what should be done.

**Response:** Comment accepted and changes have been made. Please see the highlighted manuscript line (339-345).

**Response reviewer 2**

38. The title is not correct. I should read “Heavy khat (Catha edulis) chewing and dyslipidemia as cardiovascular risk factors among hypertensive patients in Jimma, Ethiopia: Unmatched case3 control study”

**Response:** Comment accepted and changes have been made. Please see the highlighted manuscript line (1-3).

39. On page 8, under biochemical analysis you did describe the quality control processes that were undertaken to ensure the reliability of the results. How did you measure these results? Did you use an automated analyzer and what was the name of the analyzer and the country or company of manufacture?

**Response:** Comment accepted and changes have been made. Please see the highlighted manuscript line (193-195).

40. You have not stated how your independent variables were collected or have not defined them.

**Response:** Comment accepted and changes have been made. Please see the highlighted manuscript line (120-135).

41. In your data analysis on page 9, you did not state which variables were controlled for. Similarly, on page 19, line 256, you stated that possible confounders were adjusted for. What were these confounders?

**Response:** Comment accepted and changes have been made. Please see the highlighted manuscript line (208-210).

42. On page 19, line 257; you mentioned that excess alcohol consumption was associated with hypertension. The bivariate analysis in the table did not mention excess alcohol consumption. The category there is “still drinking” and this is different from excess alcohol consumption.

**Response:** Comment accepted and changes have been made. Please see Table 2 and Table 3.

43. If after adjusting for confounder’s excess alcohol consumption, educational status, vigorous work-related activity etc. in addition to Khat chewing were each independently associated with hypertension why is your focus only on Khat chewing? If your interest is on Khat chewing then you need to adjust for all other variables (confounders) that are associated hypertension before you determine whether Khat chewing is independently associated with hypertension. Again, in your conclusion you stated other factors that are associated with hypertension. Why Khat chewing should be given particular attention.

**Response:** Thanks for your valuable comment and we will accept any amendment forwarded. We selected this topic after analysis had been done and all mentioned risk factors were significantly associated to hypertension but heavy khat chewing and dyslipidemia had strong association, like 18times and 14 times, respectively. So we thought this should be the title and gave emphasis. And also as khat is cultivated in Arabian countries and east Africa, it needs further studies, so we wanted to pin point in this issue.

44. On page 7, state the reference/source for the cut off value for hypertension using the National Committee Categorization of Hypertension.

**Response:** Comment accepted and changes have been made by using American college of cardiology’s classification.

45. Define OCP in both tables 1 and 2 and COR in table 2.

**Response:** Comment accepted and changes have been made. Please see Table 2 and Table 3.

46. The statement from lines 73 to 75 under the introduction is not correct. Check this “…..and the association of the two variables and other factors to the study area and our country, Ethiopia”

**Response:** Comment accepted and changes have been made.

47. The statement in line 46 and 47 is quite ambiguous. Which percentage is for which condition?

**Response:** Comment accepted and changes have been made. Please see the highlighted manuscript from line (55-56).
