# Supplementary material for: Heavy khat (Catha edulis) chewing and dyslipidemia as modifiable hypertensive risk factors among patients in Southwest, Ethiopia: Unmatched case-control study
Source: PLoS One. 2021 Oct 26;16(10):e0259078. doi: 10.1371/journal.pone.0259078 (PMC8547649; doi:10.1371/journal.pone.0259078)
Supplement: Attachment — Submitted filename: response to reviewers.docx [file pone.0259078.s007.docx]

**Manuscript Title:** Heavy khat (Catha edulis) chewing and dyslipidemia as modifiable hypertensive risk factors among patients in Southwest, Ethiopia: Unmatched case-control study

**Dear editor and reviewers**

We would like to extend our heartfelt gratitude and appreciation for your valuable comments and priceless time. Thank you, your comments have helped us a lot to improve the manuscript and focus on important points and also a base for our future performance. We are very happy with all the points raised because this is not only a comment rather we learnt a lot. We tried to address all comments point by point in this paper.

# Responses for the editor

1. Dear editor, we tried to include all concerns raised by the reviewer in the response to the reviewer section below including measurement of key variables, data analysis, and write of the manuscript and justification for adding new authors.

# Response to reviewer 1

1. The initial submission had only a single author. However the present submission has two additional authors (totally three). The submitting author should justify if these newly included authors really deserve being listed as authors.

**Response:** Thanks for the comment and sorry for not including them in the initial submission. Of course this authors participated in the work and there specific role has been listed in the “author contribution”. A vast study had been carried out on some chronic illness and some of the data is under write up and this paper concerning hypertension is taken from it.

2. The entire manuscript still needs careful language revision. As it stands now, the language is not suitable for publication.

**Response:** Comment accepted and we tried to make changes.

3. The description in the third paragraph of the introduction (lines 65-70) is confusing and not coherent. It needs to be re-written.

**Response:** Comment accepted and amendments have been done. Please see the highlighted manuscript from line (62-85).

4. The fourth paragraph of the introduction (lines 71-79) doesn't connect smoothly to the preceding and succeeding paragraphs. Must be revised.

**Response:** Comment accepted and changes have been made. Please see the highlighted manuscript again from line (62-85).

5. In the description of the study setting, line 97, provide the URL of JUMC.

**Response:** Comment accepted and the URL of JUMC was provided. Please see the highlighted manuscript from line (94).

6. The main exposure variables of interest in the present study were "heavy khat use" and "dyslipidemia". So why was "past history of cigarette smoking" used as an exposure variable in the sample size calculation? It is not in line with the objective of the study.

**Response:** The sample size was calculated and fixed during the development of proposal. And the title was modified after analysis because heavy khat chewing and dyslipidemia have a higher odds ratio. Any further recommendation on the title can be accepted.

7. The sampling procedure still needs a clearer description.

**Response:** Comment accepted and changes have been made. Please see the highlighted manuscript from line (136-140).

8. In the initial submission, it was stated that the tool was translated to the local language in Oromia (i.e., Afaan Oromoo). In the revised submission, the authors claim that the tool was translated to Amharic, and there is no mention of the language stated in the initial submission. This casts doubt on the trustworthiness of the authors’ claims.

**Response:** Comment accepted and changes have been made and we will attach questionnaires with local languages.

9. The reason for back-translation should be revised. The reason stated as “to check for internal consistency” is not sound and convincing.

**Response:** Comment accepted and changes have been made. Please see the highlighted manuscript from line (149-151).

10. When On page 9, the authors have provided a detailed procedure for arterial blood pressure measurement. However, earlier in the manuscript, they have stated that "Cases were patients who were already diagnosed with hypertension by a physician or those taking antihypertensive drugs during the study period." If that is how cases were ascertained, how did the authors make sure that the BP measurement for cases was done as per the procedure described there? Clearly, measurement of blood pressure for cases was not under the control of the authors.

**Response:** Comment accepted and data collectors were taken from the chronic illness clinic where the same procedure was carried out. And the procedure was used for the control groups, too.

11. The reference cited in relation to the use of a p-value of 0.25 for selection of variables for the multivariable model (reference #21, page 11) does not seem to be the appropriate reference for the argument provided. The cited reference (#21) is a book 1about clinical examination and has nothing to do with statistical data analysis. The appropriate reference must be cited. Also check the appropriateness of all other citations.

**Response:** Comment accepted and changes have been made. Please see the highlighted manuscript from line (204).

12. Better to describe the results pertaining to physical activity in terms of the intensity of the physical activity. The “active/inactive” dichotomy doesn’t seem to make much sense.

**Response:** Comment accepted and modifications have been made. Please see the highlighted manuscript from line (235-236) and Table 2 and 3.

13. One of the independent variables used in the multivariable analysis was oral contraceptive use. However, oral contraceptive use applies only to females. Such a variable which applies to only a sub-set of the sample should not be used in multivariable analysis as it will significantly diminish the total sample used in the multivariable analysis. In the present manuscript, including OC use will preclude all males from the analysis.

**Response:** Comment accepted and amendment has been done. We tried to screen starting from Epi data and coding in SPSS. There was of course some gap with the analysis and even coding. But we did rigorous analysis to include all raised concerns. Please see the Table 2 and Table 3.

14. Further, the analysis of "amount of khat chewed per session" should include “non-chewers” in the analysis so that the analysis will apply to the entire sample, not to a sub-set of the sample (khat chewers) only. Hence, recode the "amount of khat chewed per session" as "non-chewer, mild khat chewer, moderate khat chewer, and heavy khat chewer" and re-do the analysis.

**Response:** Thanks for the comment and “non-chewer” has been included. Please see Table 2 & Table 3).

15. Do not state odds ratios with 95% CIs in the discussion section.

**Response:** Comment accepted and amendment has been done.

16. The results in the present study pertaining to the association of level of education with hypertension are contrary to several previous studies. The authors tried to justify this simply as “…because of the difference in the socio-economic status of the study population, cultural, dietary and lifestyle differences of the areas and countries,” (lines 287-289). What if it is due to methodological problem with the present study? The authors should re-check the analysis. Even if the re-analysis may not change the results, the argument the authors come up with must be very critical and convincing.

**Response:** Comment accepted and amendment has been done. Please see from line (280-282).

17. In lines 299-300, the authors recommend an experimental study to further investigate the association of khat chewing with hypertension-related morbidity and mortality. Is that practicable and ethically sound?

**Response:** Thanks for the comment. Though old we had included some literatures regarding the effect of khat on myocardial function and effect on blood vessels. So, further clinical trials involving animal studies can be carried out to strengthen the result (292-295).

18. Discussion of the effect of physical activity on hypertension risk (lines 304-310) must be done carefully and critically taking account of the whole body of evidence on the topic. The authors' arguments in their present form lack a critical analysis of the state-of-the-art evidence.

**Response:** Comment accepted and amendment has been done. Please see the highlighted manuscript from line (300-309).

19. In the last paragraph of the discussion, the authors need to provide careful and critical discussion of the limitations of the present study. What has been provided is very superficial and not convincing.

**Response:** Comment accepted and amendment has been done. Please see the highlighted manuscript from line (330-336).

20. Check the authors' guideline of PLoS ONE for the appropriate placement of tables within the manuscript.

**Response:** Comment accepted and changes have been made. Please see Table1-3.

21. In Tables 1 & 2, include a p-value column to show whether the differences were significant or not.

**Response:** Comment accepted and change has been made. Please see Table1&2.

22. Better to remove the p-values from Table 3 as it is unclear whether they were based on the crude analysis or the adjusted analysis. Besides, ORs with 95% CIs can tell both about the magnitude and significance of the association. Hence, the use of a p-value is superfluous. Also, include columns which provide the cross-tabulation of each independent variable with the dependent variable in Table 3.

**Response:** Comment accepted and changes have been made. Please see Table 3.
