# Supplementary material for: Heavy khat (Catha edulis) chewing and dyslipidemia as modifiable hypertensive risk factors among patients in Southwest, Ethiopia: Unmatched case-control study
Source: PLoS One. 2021 Oct 26;16(10):e0259078. doi: 10.1371/journal.pone.0259078 (PMC8547649; doi:10.1371/journal.pone.0259078)
Supplement: Attachment — Submitted filename: response to reviewers.docx [file pone.0259078.s008.docx]

**Manuscript Title:** Heavy khat (Catha edulis) chewing and dyslipidemia as modifiable hypertensive risk factors among patients in Southwest, Ethiopia: Unmatched case-control study

**Dear editor and reviewers**

We would like to extend our heartfelt gratitude and appreciation for your valuable comments and priceless time. So far we were not only being commented also we were learning a lot from your points raised. Thank you, your comments have helped us a lot to improve the manuscript and focus on important points and also a base for our future performance. We tried to address all comments point by point in this paper.

# Responses for the editor

1. Dear editor, we tried to include all concerns raised by the reviewer in the response to the reviewer section below including selection of variables for multivariable model. We already excluded the inclusion of the OCP in the multivariable analysis as per the previous comment and done the analysis again unfortunately the variable was not removed from Table 3. That was a mistake and we feel sorry for that, now it is definitely removed.
2. We also tried to follow PLOS ONE’s criteria for formatting, structuring and write up of the manuscript. Thanks for the comments.

**Response to reviewer 1**

1. The language still needs careful revision.

**Response:** Comment accepted and changes had been done.

2. The term "physical inactivity" in the abstract must be revised.

**Response:** Comment accepted and amendment has been done. Please see the highlighted manuscript line (32).

3. One of the justifications the authors provided for doing the present study in lines 78-80 is "Because previous studies conducted in Ethiopia were cross-sectional in nature which lacked a cause-and-effect relationship." The case-control design used by the present study also has the same limitation. Therefore, that argument is not convincing. It should be omitted or re-phrased.

**Response:** Comment accepted and changes have been made. Please see the highlighted manuscript again from line (78-82).

4. Better to indicate the URL of JUMC in the text describing the study setting instead of giving it a reference number and citing it as a formal reference. If it has to be cited formally, the reference information should comprise of all important information for a Web page used as a reference.

**Response:** Comment accepted and the URL of JUMC was included in the manuscript. Please see the highlighted manuscript line (91).

5. In the data collection section, the authors should state into what local languages the questionnaire was translated.

**Response:** Comment accepted and changes have been made. Please see the highlighted manuscript from line (158-159).

6. The information provided under "Arterial blood pressure measurement" (pages 9-10) should be part of the "Measurement of variables" section (page 7). Hence, merge it with the description of the "Outcome variable". Also avoid bulleted listing. Provide your arguments using complete sentences.

**Response:** Comment accepted and changes have been made. Please see the highlighted manuscript from line (112-121).

7. The response the authors provided for my earlier comment regarding measurement of blood pressure for cases is not convincing. Merely taking data collectors from the chronic illness clinic does not make the blood pressure measurement for cases similar to the blood pressure measurement of the controls.

**Response:** Cases were already selected because they were confirmed hypertensive patients OR on anti-hypertensive treatment and controls were confirmed to be free from hypertension by following the same procedure followed in the chronic illness clinics.

8. The authors have still retained OCP use in the multivariable analysis. As I commented in my previous review, OCP use applies only to females and as such should not be used as an independent variable in the multivariable model because its inclusion automatically excludes all males from the multivariable model. As it is seen from the cross tabulation in Table 3, the sample size for USP use is only 193, which is the sample size for female study participants. What the authors should clearly recognize is, in the multivariable model if the sample size for OCP use is 193, then the sample size for all other variables in the model is automatically diminished to 193. That is, 213 study participants (all males) are excluded from the analysis for all independent variables in the model. Therefore, the authors should re-do the analysis by excluding OCP use if the results of the present study are to be valid.

**Response:** We feel very sorry for not deleting the variable OCP use from “Table 3”. It was included by mistake. As per the previous comment we already removed OCP use from multivariable analysis and done the analysis again in the previous submission.

9. The authors should still check the authors' guideline of PLoS ONE for the appropriate placement of tables within the manuscript.

**Response:** Comment accepted and changes have been made. Please see “Table 1”, “Table 2” and “Table 3”.

10. The discussion still needs to be more critical.

**Response:** Comment accepted and changes have been made.

11. The authors should make sure that all references are properly written.

**Response:** Comment accepted and modifications have been made.

12. The data availability statement should be in line with PLoS ONE’s data availability policy.

**Response:** Comment accepted and changes have been made. Please see the highlighted manuscript line (358).
